# Modulating a Massive Set of Biomolecular Structures by Sono‐Mechanical Force

**DOI:** 10.1002/advs.202511687

**Published:** 2025-10-30

**Authors:** Pravin Pokhrel, Grinsun Sharma, Jaren Jenyk, Alyssa Lower, Jiahao Ji, Sajan Shakya, Joseph Haun, Hanbin Mao

**Affiliations:** ^1^ Department of Chemistry and Biochemistry Kent State University Kent OH 44242 USA; ^2^ School of Biomedical Sciences Kent State University Kent OH 44242 USA; ^3^ Twinsburg High School Twinsburg OH 44087 USA; ^4^ The College of Wooster Wooster OH 44691 USA; ^5^ Advanced Materials and Liquid Crystals Institute Kent State University Kent OH 44242 USA

**Keywords:** DNA G‐quadruplex, DNA hairpins, sono‐delivery, sono‐mechanics, ultrasound

## Abstract

Mechanical modulation of biomolecular structures by single‐molecule techniques, such as optical tweezers, has revealed subtle conformational dynamics and enabled precise modulation of functional properties. However, such tools are limited to manipulating one or a few molecules at a time in extracellular settings, posing significant challenges for scaling force‐based methods to achieve high sensitivity and efficacy both outside and within cells. Here, low‐power (<5.3 mW cm^−2^) ultrasound is employed to generate sono‐mechanical forces without formation of sonodynamic radicals, which are known to irreversibly alter molecular structures. By calibrating against optical‐tweezers‐based single‐molecule force spectroscopy, this study quantifies for the first time that at least 29 pN sono‐mechanical force can be generated at 5.3 mW cm^−2^ sonication power, capable of simultaneously and reversibly unfold an ensemble set of DNA structures, including G‐quadruplexes and hairpins. Notably, the same sono‐mechanical unfolding is observed in cells, where intercalated doxorubicin ligands are released from unfolded DNA hairpin carriers, resulting in targeted cancer cell death. These findings show ultrasound can simultaneously manipulate a large population of biomolecules without requiring fixed orientations, offering a flexible and nonintrusive force generation to reversibly unfold molecular structures. This platform holds profound potential for applications in molecular biophysics, smart materials, and precision medicines.

## Graphical abstract

Using low‐power ultrasound exposure, picoNewton forces are generated to release loaded drugs after unfolding secondary structures in DNA nanoassemblies. This opens a door to reversibly manipulate a large set of biomolecular structures both inside and outside cells without the damages incurred by high‐power sonications currently used in the field.

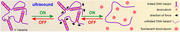

## 1 Introduction

Biomacromolecules such as proteins and nucleic acids exhibit dynamic conformations essential for numerous biological processes.^[^ ^1^ ^]^ These structures are sensitive to external forces, which can modulate their functions by inducing transitions between folded and unfolded states. Conventional force‐based approaches for manipulating biomolecular structures include optical tweezers, magnetic tweezers, and atomic force microscopy, which provide mechanochemical information at the single‐molecule level.^[^ ^2^ ^]^ However, these techniques are limited in their ability to manipulate a large population of biomolecules simultaneously, restricting their application only in low‐throughput, small‐scale studies.

At the bulk level, hydrodynamic shear has been employed to mechanically manipulate a large set of biomacromolecules by leveraging fluid flow, such as shearing^[^ ^3^ ^]^ and centrifugation.^[^ ^4^ ^]^ However, these approaches present significant limitations, particularly in biological systems. For example, high centrifugal and shear forces can compromise cell integrity, leading to membrane disruption and intracellular damage.^[^ ^5^ ^]^ As a result, there is a lack of method to quantitatively manipulate force on a large ensemble of molecules inside cells simultaneously.^[^ ^6^, ^7^, ^8^ ^]^ Furthermore, large or elongated macromolecules experience excessive tensile forces, which can easily surpass their mechanical stability, resulting in irreversible fragmentation and loss of native functionality.^[^ ^9^ ^]^ These drawbacks highlight the need for alternative strategies that enable non‐intrusive and reversible mechanical manipulation on a population of molecules without compromising cell viability.

Ultrasound‐mediated mechanical manipulation offers a rather efficient and non‐invasive alternative for biomolecules at an ensemble level.^[^ ^10^, ^11^, ^12^, ^13^, ^14^, ^15^, ^16^ ^]^ Unlike single‐molecule mechanical techniques that require specialized instrumentation, ultrasound can exert mechanical stress on a population of biomolecules simultaneously, facilitating functional modulation of molecules on a large scale.^[^ ^12^ ^]^ One of the key advantages of ultrasound is its biocompatibility, as cells can tolerate significant ultrasonic exposure without noticeable loss of viability or function.^[^ ^10^ ^]^ This makes ultrasound particularly suitable for studying biomolecular dynamics under physiological conditions. Mechanical shear forces can be generated by ultrasound through acoustic cavitation, radiation pressure, and microstreaming.^[^ ^16^ ^]^ However, most ultrasound applications used high sonication powers to generate strong shear forces and produce radicals, which respectively lead to irreversible breakage of covalent bonds and modification of chemical structures in molecules.^[^ ^12^, ^13^, ^17^, ^18^, ^19^, ^20^, ^21^ ^]^ We reasoned that by fine‐tuning parameters such as frequency, intensity, and exposure duration, ultrasound can enable targeted, tunable, and reversible mechanical unfolding of a large set of molecular structures without the damaging effect from sonodynamic radical formation.^[^ ^13^ ^]^ Reversible unfolding enables repeated probing of the same targets, providing long‐term and well‐controlled payload release in therapeutic applications.^[^ ^22^, ^23^ ^]^ Importantly, operating in a reversible, non‐destructive force regime enhances safety and biocompatibility by avoiding collateral damage, which is a critical consideration for translational applications. Taken together, these advantages position ultrasound as an unprecedented tool for high‐throughput investigation of molecular biophysics, large scale preparation of responsive materials, and noninvasive implementation of targeted therapeutics.

In this study, we pioneered ultrasound‐generated mechanical forces to unfold DNA secondary structures such as hairpins and G‐quadruplexes. To increase the sensitivity of the force response, we embedded these structures in a single‐stranded DNA (ssDNA) scaffold using Rolling Circle Amplification (RCA).^[^ ^24^, ^25^ ^]^ Application of ultrasound induced mechanical stress (i.e., sono‐mechanical force) led to reversible unfolding of DNA hairpins and G‐quadruplexes. After comparing with the mechanical unfolding of the same structures using optical tweezers, we revealed that an ultrasound power of 5.3 mW cm^−2^ produced force ≈29 pN on an ssDNA of >10 kb in length. To demonstrate that the sono‐mechanical force can be applied to biomolecules inside cells, we introduced cancer‐cell targeting AS1411 aptamers in the tandem array of DNA hairpins preloaded with an anticancer drug doxorubicin.^[^ ^26^ ^]^ We found that a 15 s sonication on cancer cells (HeLa) internalized with this DNA construct significantly reduced cell viabilities by releasing the doxorubicin due to the sono‐mechanical unfolding of the DNA hairpin carriers inside cells. In summary, we have 1) demonstrated operation of ultrasound in a low‐power, non‐radical producing regime, which probes purely mechanical, non‐covalent unfolding of nucleic acid secondary structures; 2) offered a first direct quantification of sono‐mechanical force generated during ultrasonification; and 3) demonstrated simultaneous reversible unfolding of a ensemble set of DNA secondary structures with the release of loaded drugs to target cancer cells. Collectively, these results highlight ultrasound as a powerful platform for in situ mechanical manipulation of biomolecules both within and outside cells. The long‐range and non‐invasive ability to exert picoNewton‐level mechanical forces on a massive set of molecular ensembles opens new avenues for simultaneously and reversibly modulating numerous mechanochemistry and mechanobiology processes.

## 2 Results and Discussion

### 2.1 Unfolding an Array of DNA Hairpins by Ultrasound

**Figure 1:**
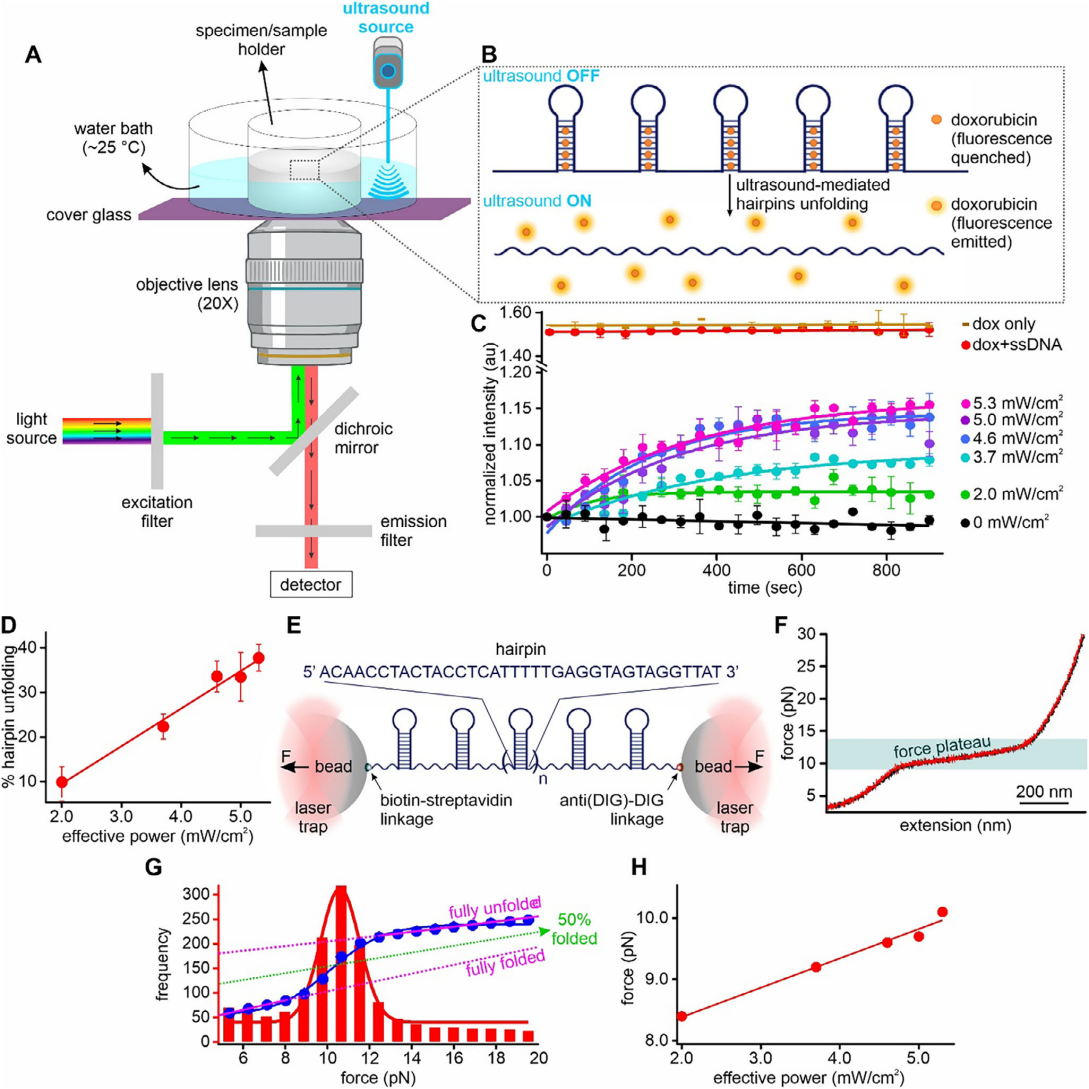
Ultrasound‐mediated unfolding of DNA hairpin structures. A) Schematic of the sono‐mechanical experiment. B) Schematic of ultrasound‐mediated unfolding of a DNA‐hairpin array (average length >10 kb). When sonication is on, doxorubicin molecules (2 µm) are released after DNA hairpins in RCA construct (1 µm) are unfolded, resulting in C) fluorescence increase at different ultrasound powers at 25 °C. Temperature was maintained using a controlled temperature water bath around the sample holder. Brown and red data, respectively, represent 2 µm doxorubicin (dox) only and 2 µm dox mixed with 1 µm non‐structural forming random ssDNA RCA (see Table S1, Supporting Information, for detailed sequence) at 5.3 mW cm^−2^ sonication power (see Section S2, Supporting Information, for calculation of ultrasound power). Solid curves depict either linear or exponential fittings. D) % hairpin unfolding versus average ultrasound power obtained from the sono‐mechanical experiment in (C). The data are linearly fit to guide eyes. E) Optical tweezers set up for the single‐molecule force spectroscopy of the same ssDNA construct containing an array of hairpin structures. Horizontal arrows depict direction of mechanical forces. F) Typical force‐extension (FX) curves obtained from stretching (red) and relaxing (black) the single‐molecule RCA hairpin construct (see Section S3, Supporting Information, for synthesis of RCA products). G) Reconstructed force histogram (red) and cumulative unfolding percentage of hairpin structures in the RCA construct (blue). Solid red curve depicts Gaussian fitting, while the two dotted pink lines represent the extrapolations depicting 100% and 0% folded hairpins. Green dotted line depicts 50% folded hairpins (see Section S16, Supporting Information, for details). H) Calibration of sono‐mechanical force against sonication power. The data are linearly fit to guide eyes. Error bars depict standard deviations from at least three independent measurements.

We used an ultrasonicator device (Digital Sonifier 450, Branson Ultrasonics Corporation) with a cylindrical microtip transducer probe (diameter: 3 mm) on an inverted fluorescence microscope (Figure **1**A) for real‐time observations of the sonication effect on a hairpin, 5′ AAC CTA CTA CCT CAT TTT TGA GGT AGT AGG TT 3′ (the underline indicates the loop). We used doxorubicin to monitor the unfolding process: when intercalated into the duplex DNA hairpin stem, the doxorubicin's fluorescence is quenched.^[^ ^27^, ^28^, ^29^ ^]^ We found that the single‐unit DNA hairpin did not unfold under 20 kHz ultrasound at sonication power up to 5.3 mW cm^−2^ (Section S7 and Figure S10, Supporting Information), beyond which fluorescence images could not be captured stably due to the vibration generated by the ultrasound. The failure to unfold single‐unit DNA hairpin is likely due to the fact that a short polymer (such as the single‐unit hairpin) has insufficient chain length to experience significant hydrodynamic shear,^[^ ^30^, ^31^ ^]^ and thus experiencing much lower intramolecular tension under applied sonication power. In contrast, a longer construct converts distributed hydrodynamic shear into a larger tension exerted on the structural motifs along the chain. Therefore, to apply a force that is sufficient to unfold the hairpin, we reasoned that increasing the length of the DNA hairpin‐containing strand would enable higher mechanical force to be applied to the hairpins, thereby promoting unfolding.^[^ ^32^, ^33^, ^34^ ^]^

To test this, we synthesized a long ssDNA (length >10 kb) that consists of a tandem array of the same 32‐nt hairpin‐forming sequence via the RCA protocol (Section S3 and Figure S1, Supporting Information, for details).^[^ ^24^, ^35^, ^36^ ^]^ We first designed a linear ssDNA (*hairpin RCA template*, 56‐nts, Table S1, Supporting Information, for sequence) consisting of a sequence complementary to the hairpin‐forming sequence. A short oligonucleotide (*splint*, 42‐nts, Table S1, Supporting Information, for sequence) was used to circularize this linear ssDNA by splint ligation.^[^ ^37^, ^38^ ^]^ The same *splint* served as the primer to initiate the RCA reaction, forming a long ssDNA with a tandem array of hairpin forming sequences. The formation of hairpins on the RCA product was confirmed by the optical‐tweezers experiments^[^ ^25^, ^36^ ^]^ (Figure 1E,F; Section S4, Supporting Information, for details).

To investigate the ultrasound‐mediated mechanical unfolding of the hairpin repeats in the RCA construct, we used the same doxorubicin‐based fluorescence imaging as mentioned in Figure 1A. The 2 µm doxorubicin was first quenched in presence of an RCA construct with an effective 1 µm hairpin concentration, which has been established through titration of doxorubicin against the hairpin RCA (Section S12 and Figure S14, Supporting Information, for details). Upon 2.0–5.3 mW cm^−2^ sonications, the hairpins were unfolded, releasing doxorubicin with increased fluorescence intensity (Figure 1B,C). Previously, most studies have used much higher acoustic intensities in the range of 0.1–10 W cm^−2^.^[^ ^12^, ^13^, ^39^ ^]^ This generated strong shear forces^[^ ^12^, ^40^ ^]^ and produced radicals,^[^ ^13^, ^41^ ^]^ which respectively led to irreversible breakage of covalent bonds and modification of chemical structures in molecules. It is noteworthy that even at the maximum power of 5.3 mW cm^−2^ used here, the RCA construct stayed intact (see gel image in Section S6 and Figure S9, Supporting Information). The rate of the fluorescence signal increase was dependent on the ultrasound power (Figure 1C), which was consistent with the fact that the higher the ultrasound power, the more the ultrasound force, and the faster the unfolding rate of the hairpin structures. After 10 min of sonication, the fluorescence intensity reached a plateau (Figure 1C) at specific ultrasound power, indicating that a steady state was obtained where the folding and unfolding rates of hairpins reached equilibrium. When sonication was turned off, a sharp decrease in fluorescence was observed within 5 min (Figure S18, Supporting Information). This suggests the refolding of the hairpins, allowing doxorubicin to rebind to their duplex stems. This observation gives a strong support that the RCA construct remains structurally intact under 5.3 mW cm^−2^ ultrasound exposure for 15 min. Previous studies have indicated that under the same 20 kHz sonications, shear force is much more predominate than the radical formation,^[^ ^42^, ^43^ ^]^ which may alter chemical structures in DNA. Consistent with these findings, we observed identical sono‐mechanical unfolding of the RCA construct in the presence of 0.05% DMSO (Figure S19, Supporting Information), a known radical scavenger.^[^ ^44^ ^]^ This strongly supports that hairpin unfolding is predominantly driven by mechanical shear force rather than free radical‐induced structural change.

In a control experiment (− control) without sonication, no change in fluorescence intensity was observed over 15 min (Figure 1C, black). This suggests that no hairpin was unfolded without ultrasound, which served as the lower boundary for hairpin unfolding (i.e., 0%). Similarly, in a sample (+ control) in which the hairpin forming sequence was replaced by non‐structure forming random ssDNA sequence (Table S1 for the sequence and Figure S2, Supporting Information, for details) (Figure 1C, red), fluorescence intensity of doxorubicin remained unchanged at an elevated level when 5.3 mW cm^−2^ ultrasound was applied. This was because the doxorubicin didn't bind tightly to the random ssDNA sequence to quench its fluorescence. In fact, such fluorescence intensity was only slightly lower than that of the free doxorubicin at 5.3 mW cm^−2^ ultrasound power (Figure 1C, “dox only”). Therefore, this fluorescence intensity level sets the boundary for the maximally achievable intensity after all hairpins are unfolded in the RCA construct (i.e., 100% unfolding). It is noteworthy that due to equilibrated hairpin unfolding and refolding processes, the plateaus of the fluorescence intensity never reached the maximum level (Figure 1C, red) even at the maximum ultrasound power of 5.3 mW cm^−2^. Therefore, we used average fluorescence intensities of the positive and negative controls to respectively represent 100% and 0% hairpin unfolding, from which % hairpin unfolding at specific ultrasound power was determined at respective plateau levels (Figure 1D).

### 2.2 Sono‐Mechanical Force Calibration

To calibrate the sono‐mechanical force exerted on DNA structures at a specific ultrasound power, we compared the percentage of hairpin structures unfolded by ultrasound with that unfolded by mechanical force using optical tweezers in the same buffer (10 mm Tris, 100 mm KCl, pH 7.4) at room temperature.^[^ ^3^ ^]^ To this end, we labelled a >10kb‐long hairpin‐forming ssDNA construct with biotin at the 5′ end and digoxigenin (DIG) at the 3′ end (Section S4 and Figure S6, Supporting Information, for details). These modifications facilitated the tethering of the construct between a pair of optically trapped beads via biotin‐streptavidin and anti(DIG)‐DIG affinity linkages (Figure 1E), respectively. Tension was then applied to the tethered construct by moving the two trapped beads apart using a mirror that steered one of the trapping lasers (Section S4, Supporting Information, for details).^[^ ^45^ ^]^ This allowed mechanical unfolding of the hairpin structures formed in the DNA construct.

When the ssDNA construct was stretched, a force plateau appeared in the force‐extension (FX) curves (Figure 1F, red curve), indicating simultaneous unfolding of hairpin structures in the construct under tension.^[^ ^25^, ^36^ ^]^ During relaxation, the FX curve closely followed the same trajectory as the stretching (Figure 1F, black), indicating rapid unfolding and refolding kinetics of DNA hairpins.^[^ ^46^ ^]^ The force plateau in the FX curves is a hallmark of the unfolding of a tandem array of hairpin structures in a single‐molecule DNA construct.^[^ ^25^, ^36^ ^]^ When the histogram of stretched force along the FX curves was plotted, a distinct population with a Gaussian center at 10.5 pN was observed, indicating the most probable force to unfolding hairpin arrays (Figure 1G, red). After integration of this unfolding force histogram, we obtained the cumulative unfolding percentage versus mechanical force (Figure 1G, blue, Section S16, Supporting Information, for details). Assuming that the percentage unfolding of the hairpin structures was the same between the single‐molecule mechanical unfolding (Figure 1G) and the sono‐mechanical unfolding (Figure 1C) at the same force, we calibrated the mechanical force versus ultrasound power (Figure 1H), which depicted that at least 10.1 pN could be applied to the hairpin forming RCA construct (> 10 kb) at 5.3 mW cm^−2^ sonication power. This calibration curve was then used to estimate the force experienced by RCA constructs with shorter lengths (Section S8, Supporting Information, for details). We found that 9.6 pN and 8.6 pN were experienced in the 1.5 kb (range 1–10 kb) and 0.6 kb (range 0.5–1 kb) RCA constructs, respectively, (Section S8 and Figure S11, Supporting Information) at the 5.3 mW cm^−2^ sonication power. These results strongly supported that longer strands could experience higher force^[^ ^32^ ^]^ at a fixed sonication power.

### 2.3 Sono‐Mechanical Modulation of other DNA Secondary Structures

**Figure 2:**
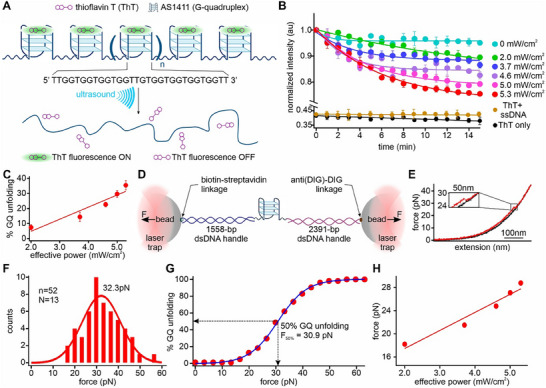
Sono‐mechanical force modulates DNA secondary structures. A) Schematic of sono‐mechanical unfolding of an AS1411 G‐quadruplex (GQ) array. B) Change in fluorescence intensity of the 2 µm thioflavin T (ThT) bound GQ‐containing RCA construct (length >10 kb, 1 µm) over time under different ultrasound powers at 25 °C. Temperature was maintained using a temperature controlled water bath around the sample holder. When sonication is on, the GQ‐ThT fluorescence decreases as ThT dissociates from unfolded GQ. Black and brown data, respectively, represent 2 µm ThT only and 2 µm ThT mixed with 1 µm effective concentration of the ssDNA contained in a non‐structural forming RCA construct (Table S1 and Figure S2, Supporting Information, for details) at 5.3 mW cm^−2^ sonication power. Solid curves depict either linear or exponential fittings. C) %GQ unfolding versus sonication power obtained from sono‐mechanical experiment in (B). Solid line depicts a linear fit. D) Optical tweezers set up for the single‐molecule force spectroscopy of the AS1411 G‐quadruplex. Horizontal arrows depict direction of mechanical forces. E) Typical force‐extension (FX) curves obtained from the stretching (red) and relaxing (black) of a single‐molecule AS1411‐GQ construct. Blow up image represents a rupture event suggesting the unfolding of an AS1411 GQ. F) Unfolding force histogram of the AS1411‐GQ structure. Solid red curve depicts a Gaussian fitting. G) Cumulative %unfolding of the AS1411‐GQ obtained from the unfolding force histogram in (F). The blue curve is a sigmoidal fit. (H) Calibration of the forces experienced in >10 kb GQ‐containing RCA construct under different sonication powers. Solid line represents a linear fit. Error bars depict standard deviations from at least three independent measurements.

To test that sono‐mechanical modulation is a generic structural modulation approach, we used a more stable DNA secondary structure, G‐quadruplex (GQ), which has unfolding and refolding kinetics slower than DNA hairpins.^[^ ^47^, ^48^ ^]^ G‐quadruplexes are stable secondary structures formed in guanine‐rich DNA sequences, where four guanine bases are associated through Hoogsteen hydrogen bonding to form a planar tetrad.^[^ ^49^, ^50^ ^]^ Several tetrads stack with each other, which is further stabilized by monovalent cations, such as potassium or sodium, by binding to the central cavity of the tetrads. Given that AS1411 G‐quadruplex^[^ ^51^ ^]^ can enter cells after binding to the nucleolins overexpressed on cancer cell surface,^[^ ^52^ ^]^ this structure has been used for cell delivery purposes after attachment of payload molecules. We first investigated the sono‐mechanical effect on a tandem array of AS1411 GQ in a >10 kb ssDNA prepared by RCA (Section S3 and Figure S3, Supporting Information, for details). To monitor the folding or unfolding of the AS1411 GQ, we utilized a fluorogenic dye Thioflavin‐T (ThT), which fluoresces upon binding to folded GQ.^[^ ^53^, ^54^ ^]^ We hypothesized that ultrasound would unfold GQ structures formed in the AS1411 sequences, leading to the release of bound ThT with concomitant decrease in fluorescence (Figure **2**A). This was indeed observed during 15 min of sonication (Figure 2B). When we increased sonication power, more GQs were unfolded (Figure 2C).

To quantify the force generated by the ultrasound, we performed single‐molecule mechanical unfolding on an AS1411 GQ structure using an optical tweezers instrument. Similar to the procedure described above, we tethered a DNA construct that contained an AS1411 sequence between two optically trapped beads (Figure 2D; see Figure S7, Supporting Information, for details). During the force‐ramp experiment in optical tweezers, stretching of the DNA construct revealed distinct structural unfolding features in the force‐extension (FX) curves (red curves, Figure 2E), which indicated the prior formation of a DNA secondary structure in the single‐molecule construct. When we measured the change‐in‐contour‐length (∆*L*) (Section S5 and Figure S8, Supporting Information, for details), we found that measured ∆*L* closely matched with the predicted value for the unfolding of the AS1411 GQ (9.2 vs 9.4 nm), suggesting that the rupture feature corresponded to the unfolding of the AS1411 GQ. The formation of the GQ in the AS1411 sequence was further confirmed by circular dichroism (Section S14 and Figure S15, Supporting Information). After integrating the rupture force histogram (Figure 2F), we obtained cumulative % unfolding of the AS1411‐GQ (Figure 2G). By correlating sonication‐assisted GQ unfolding percentages (Figure 2C) with those obtained by mechanical force (Figure 2G), we estimated the forces experienced in the tandem AS1411‐GQ construct (>10 kb) at different sonication powers (Figure 2H). Our analysis revealed that the maximum force experienced by the AS1411‐GQ RCA construct was up to 28.8 pN at the maximal sonication power of 5.3 mW cm^−2^.

### 2.4 Sono‐Mechanical Force on Biomolecular Structures Inside Cells

**Figure 3:**
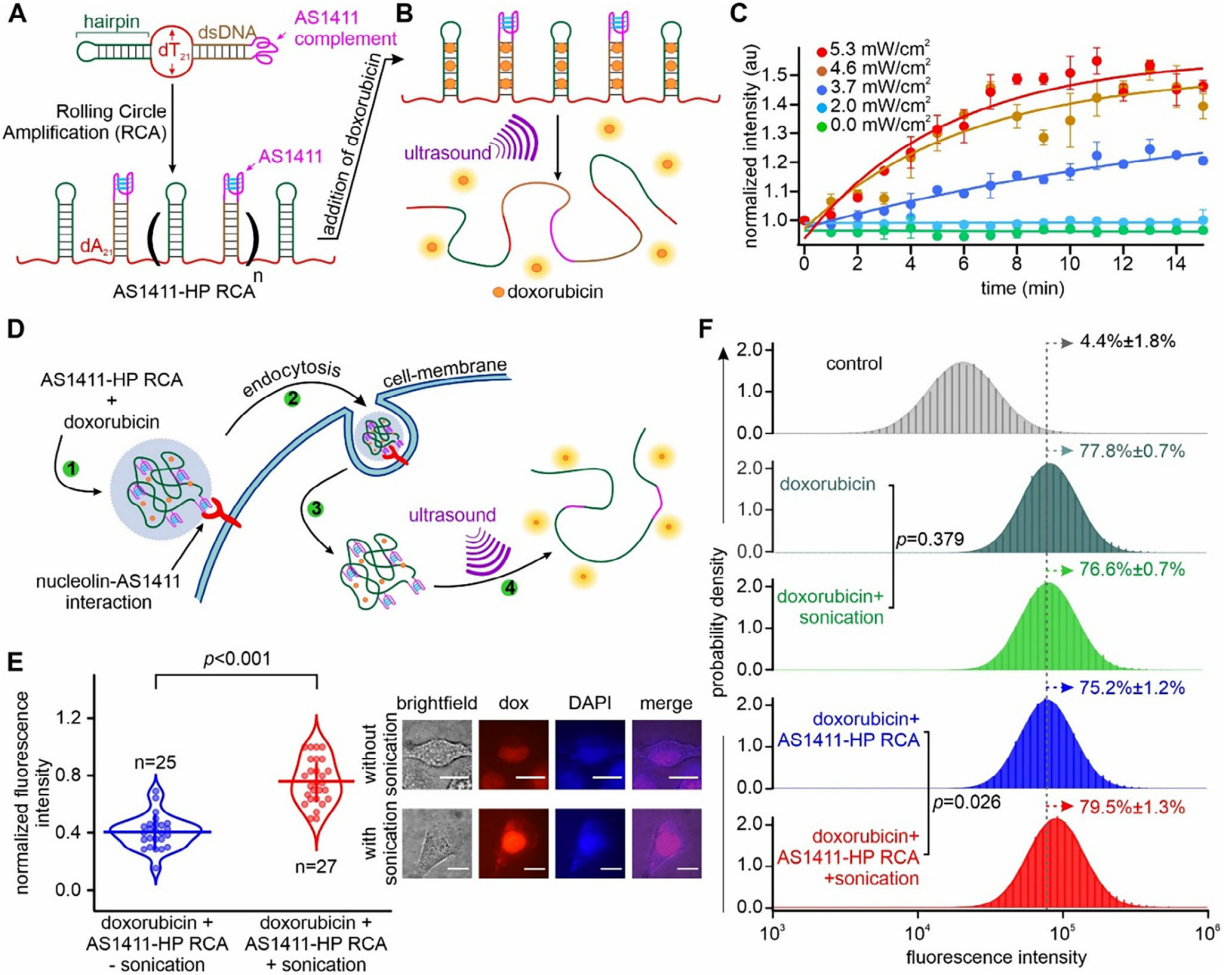
Ultrasound‐mediated unfolding of DNA hairpins in HeLa cells. A) Synthesis of tandem repeats of AS1411‐hairpin in an RCA construct (AS1411‐HP RCA) to investigate intracellular sono‐mechanical effects. B) Schematic of ultrasound‐mediated unfolding of the DNA secondary structures, resulting in doxorubicin release. C) Release of doxorubicin (2 µm) from the AS1411‐HP RCA construct (>10 kb, 1 µm) under different ultrasound powers increases fluorescence intensity. Solid curves depict either linear or exponential fittings. D) Schematic of the ultrasound‐mediated doxorubicin delivery in HeLa cells using the AS1411‐HP RCA construct. E) Violin plots of fluorescence intensity of cells treated with 2 µm doxorubicin‐bound AS1411‐HP RCA (1 µm) with (red) and without (blue) 0.46 W cm^−2^ sonication for 15 s. Horizontal and vertical lines represent mean fluorescence intensities and standard deviations, respectively. Right panels show representative brightfield and fluorescence images of single cells. The Dox channel has 532 nm excitation and 590 nm emission for detection of doxorubicin fluorescence. The DAPI channel has 350–380 nm excitation and 420–480 nm emission for imaging DAPI‐stained cell nuclei (Section S19, Supporting Information, for details). Colocalization of Dox and DAPI signals suggests doxorubicin is enriched in cell nuclei. Numbers of measured cells are marked as “n”. Scale bars: 5 µm. F) Flow cytometry analyses of HeLa cells treated with (2 µm) doxorubicin and doxorubicin‐bound AS1411‐HP RCA constructs (37.5 nm) without and with 0.46 W cm^−2^ ultrasound for 15 s. Three independently grown HeLa cells were analyzed. Error bars depict standard deviations from at least three independent measurements.

To demonstrate that sono‐mechanical force can be applied on biomolecules inside cells, we incorporated a DNA hairpin‐forming sequence to carry doxorubicin and an AS1411 sequence to facilitate cell entrance via nucleolin binding on cancer cell membranes^[^ ^55^ ^]^ (Figure **3**A; Section S3 and Figure S4, Supporting Information, for details). Such an ssDNA (“AS1411‐HP RCA”) with >10 kb in length was prepared following the RCA process (Section S3 and Figure S4, Supporting Information, for details). To assess the efficacy of sono‐mechanical unfolding of the DNA hairpin structures in this RCA construct, we first performed sonication experiments in vitro using the setup described in Figure 3B. As anticipated, sonication successfully unfolded the duplex DNA regions in the AS1411‐HP RCA constructs (Figure 3C), releasing bound doxorubicin with increased fluorescence intensity.

Next, we evaluated cellular delivery of the AS1411‐HP RCA constructs (Figure 3D). We loaded doxorubicin (2 µm) onto the AS1411‐HP RCA constructs (37.5 nm) at concentrations determined by ≈80% cell viability when RCA construct and doxorubicin were separately applied (Section S9 and Figure S12, Supporting Information, for details). It is noteworthy that under these two concentrations, all AS1411‐HP RCA constructs are expected to be completely loaded with doxorubicin molecules (Section S13, Supporting Information, for detailed calculation). The doxorubicin‐loaded AS1411‐HP RCA construct was incubated with HeLa cells, allowing internalization over a 1 h period, which is expected to complete AS1411‐mediated endocytosis.^[^ ^56^ ^]^ Indeed, single‐cell fluorescence analysis revealed that doxorubicin rapidly entered the cells, with significant (82.5%) uptake within the first hour of treatment (Section S10 and Figure S13, Supporting Information, for details). The uninternalized doxorubicin‐loaded AS1411‐HP RCA was washed off and the incubated cells were subjected to a sonication bath for 15 s at 0.46 W cm^−2^ ultrasound power (Section S2, Supporting Information, for calculation of ultrasound power). Importantly, the RCA remained intact under this sonication condition (see Figure S9, Supporting Information). Any power below 0.46 W cm^−2^ was insufficient to induce mechanical unfolding of the hairpin cargo inside cells, while higher power levels exceeded the tolerance threshold of cells and led to unwanted cell death. After sonication, we observed a significant increase (*p *<0.001) in single‐cell fluorescence intensity, which was ascribed to the release of doxorubicin from internalized AS1411‐HP RCA (Figure 3E). As controls, fluorescence intensities were not significantly different (*p* = 0.31) between doxorubicin‐treated cells with and without ultrasound (Section S15 and Figure S16, Supporting Information). Moreover, these fluorescent intensities are significantly lower than that of sonication‐treated cells incubated with doxorubicin‐loaded AS1411‐HP RCA (*p* = 0.0039), confirming the facilitated cell entrance of doxorubicin‐loaded AS1411‐HP RCA construct.

Flow cytometry was then conducted to investigate the effect of ultrasound on the intracellular release of doxorubicin (Section S11, Supporting Information, for details).^[^ ^57^, ^58^ ^]^ Two sets of cell plates were prepared. Each set included untreated cells as a control, cells‐treated with 2 µm doxorubicin, and cells‐treated with a combination of 2 µm doxorubicin and 37.5 nm AS1411‐HP RCA for 1 h. In one set, ultrasound was applied for 15 s sonication bath at 0.46 W cm^−2^ power, while the other set was not subjected to ultrasound. Analysis revealed a significant increase in fluorescence intensity in cells‐treated with doxorubicin (2nd panel, Figure 3F) or the doxorubicin‐loaded AS1411‐HP RCA constructs (4th panel) compared to untreated control cells (*p* <0.0001, 1st panel, Figure 3F). This result aligned with single‐cell fluorescence assays, where untreated cells exhibited little fluorescence, while treated cells showed nearly double the fluorescence intensity (Section S10 and Figure S13, Supporting Information). Importantly, fluorescence intensities between cells treated with doxorubicin alone (2nd panel) and those treated with the doxorubicin‐loaded AS1411‐HP RCA construct (4th panel) were not significantly different (*p *>0.05, Figure 3F), which is also consistent with the single‐cell fluorescence assay (Section S10 and Figure S13, Supporting Information). When ultrasound was applied to doxorubicin‐treated cells (3rd panel), no significant change in fluorescence intensity was observed with respect to those without ultrasound (*p* = 0.379, 2nd panel, Figure 3F. The same trend was also observed in single cell fluorescence in Figure S16, Supporting Information). This suggests doxorubicin, if bound with genomic DNA, would not get released by ultrasound, likely due to the more difficult shearing orientation^[^ ^59^, ^60^ ^]^ of genomic DNA with respect to the DNA hairpins in the RCA construct, which is subject to the sono‐mechanical force along an easier unzipping direction. Indeed, when the cells that were treated with the doxorubicin‐loaded AS1411‐HP RCA construct were subjected to 0.46 W cm^−2^ ultrasound for 15 s, significant increase in fluorescence intensity (*p* = 0.026) was observed (compare 4th and 5th panels in Figure 3F), suggesting the release of doxorubicin due to sono‐mechanical unfolding of DNA hairpin structures inside cells. This data is again consistent with the single‐cell fluorescence assay depicted in Figure 3E.

**Figure 4:**
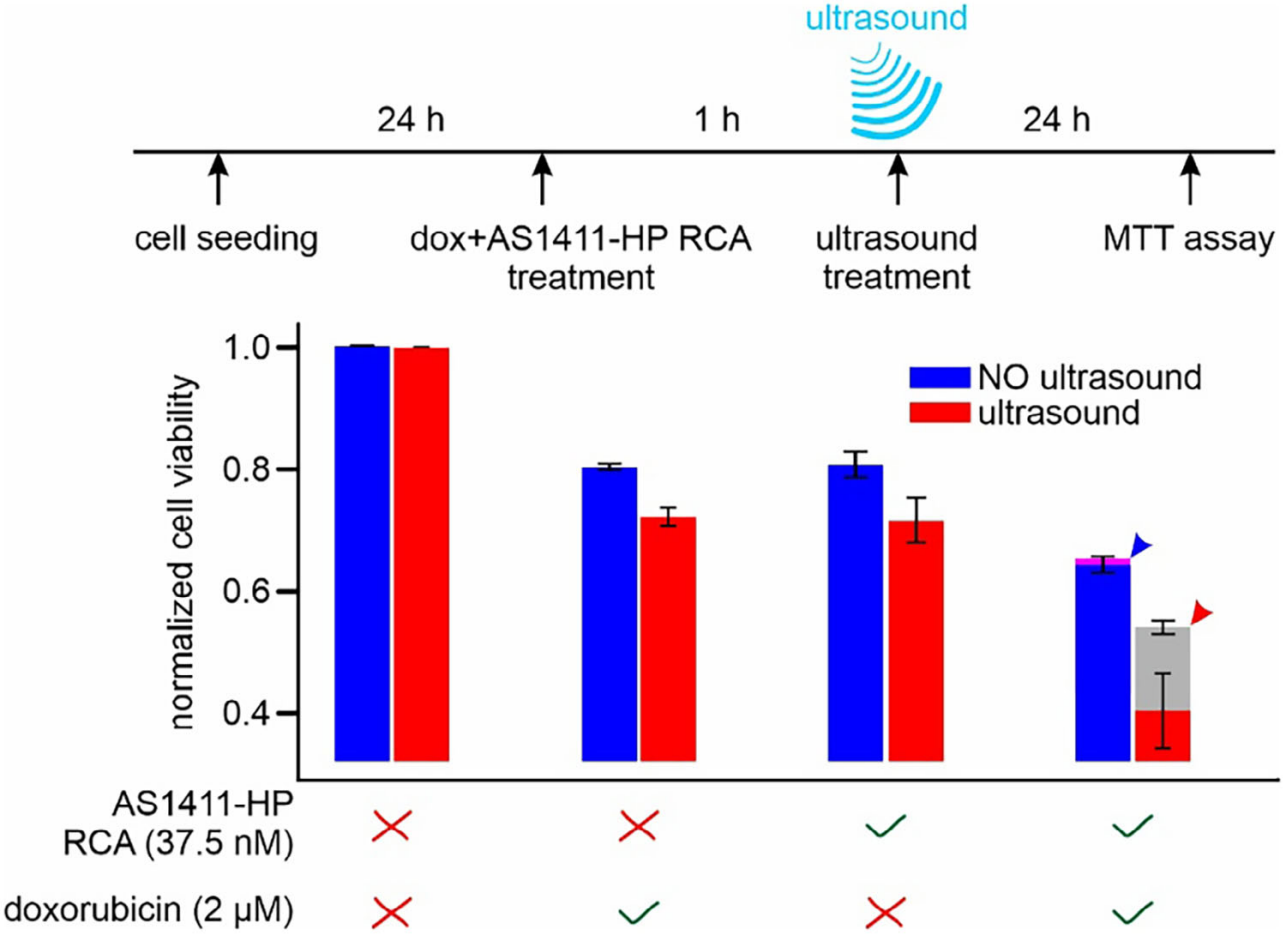
Sono‐delivery of doxorubicin in HeLa cells. MTT cell‐viability assay of ultrasound‐treated HeLa cells was performed in the presence of doxorubicin‐loaded AS1411‐HP RCA (37.5 nm) and/or doxorubicin (2 µm). Standard deviations were obtained from three independent biological repeats, each measured by six aliquots. The expected additive effects of doxorubicin and AS1411‐HP RCA on cell viability with and without 0.46 W/cm^2^ ultrasound for 15 s are respectively depicted by the two arrowheads in the last set of bar diagrams.

To confirm that doxorubicin was indeed released by sono‐mechanical unfolding of DNA structures inside cells, we used an MTT assay^[^ ^61^, ^62^ ^]^ to evaluate whether viability of HeLa cells was reduced due to released doxorubicin, an anticancer drug.^[^ ^26^ ^]^ To this end, we compared the effects of AS1411‐HP RCA, doxorubicin, their combination, and ultrasound treatment on the viability of HeLa cells (Figure **4**) (Section S9 and Figure S12, Supporting Information, for details).^[^ ^61^ ^]^ HeLa cells were first seeded in a 96‐well plate and allowed to adhere for 24 h. Subsequently, the wells were treated with 37.5 nm AS1411‐HP RCA alone, 2 µm doxorubicin alone, or their mixture. Following a 1 h incubation period, cells were washed to remove uninternalized AS1411‐HP RCA or doxorubicin. One set of treated cells was exposed to 0.46 W cm^−2^ ultrasound for 15 s, while another without. The cells were then incubated for an additional 24 h prior to performing the MTT assay.

The data collected from triplicate experiments revealed that AS1411‐HP RCA and doxorubicin individually exhibited small cytotoxic effects, with cell viability remaining above 80% without ultrasound (Figure 4). The application of ultrasound to these treatments resulted in a slight decrease in cell viability, likely due to the enhanced membrane permeability caused by ultrasound‐induced poration. This increased membrane permeability potentially facilitated greater uptake of AS1411‐HP RCA and doxorubicin, leading to marginally increased cell death. When cells were treated with a combination of AS1411‐HP RCA and doxorubicin without ultrasound, a more pronounced cytotoxic effect was observed, with cell viability decreasing to ≈65%. This value aligns closely with the theoretically calculated combined effect of RCA and doxorubicin (80.2% × 80.6% = 64.6%, indicated by a blue arrowhead in Figure 4). However, when ultrasound was applied to the combination treatment, a substantial reduction in cell viability was observed, decreasing from 64.6% to 41%. Notably, this cell viability was significantly lower (*p *<0.01) than the theoretical additive effect of AS1411‐HP RCA and doxorubicin with ultrasound (indicated by a red arrowhead in the gray bar, 72.3% × 71.3% = 51.8%).

These results collectively demonstrated that sono‐mechanical force can be successfully applied intracellularly to unfold DNA hairpin structures, thereby releasing RCA‐bound doxorubicin for subsequent DNA damage and cytotoxic effects. The synergy between AS1411‐HP RCA, doxorubicin, and ultrasound offers a promising sono‐delivery strategy for potential therapeutics in cancer treatment.

## 3 Conclusion

We have successfully employed low‐power sonication to unfold biomolecular structures without requiring fixed orientations and without the damaging effect from sonodynamic radical formation. This method paves the way to apply mechanical force on a large ensemble of molecules, a task extremely challenging to achieve by single‐molecule force manipulations. By calibrating the sono‐mechanical force against optical‐tweezers, we quantified the force generated by the ultrasound for the first time, which revealed that longer DNA templates experience larger forces. Furthermore, we have shown this sono‐mechanical modulation could unfold biomolecular structures inside cells, leading to a new type of sono‐delivery of drugs to kill cancer cells. Our approach leverages the non‐invasive nature of ultrasound, a characteristic highly valued in clinical settings due to its deep tissue penetration, precise localization, and low side effects. Therefore, this platform provides an unprecedented contribution to the fields of sono‐mechanics and sono‐chemotherapy, opening new avenues for long‐range, in‐situ manipulation of molecular structural dynamics, which can not only alter various cellular processes but also lead to responsive materials for drug delivery and precision therapeutics with much increased efficacies.

## Conflict of Interest

The authors declare no conflict of interest.

## Supporting information



Supporting Information

## Data Availability

The data that support the findings of this study are available in the supplementary material of this article.
