# Supplementary material for: Modulating a Massive Set of Biomolecular Structures by Sono‐Mechanical Force
Source: Adv Sci (Weinh). 2025 Oct 30;13(3):e11687. doi: 10.1002/advs.202511687 (PMC12806404; doi:10.1002/advs.202511687)
Supplement: Supplementary file 1 — Supporting Information [file ADVS-13-e11687-s001.pdf]

Supporting information

for

Modulating a massive set of biomolecular structures by sono-mechanical force

Pravin Pokhrel, [1,†] *Grinsun Sharma,* [2,†] *Jaren Jenyk,* [1,3] *Alyssa Lower,* [1,4] *Jiahao Ji,* [1] *Sajan Shakya,* [1] *Joseph Haun,* [1] *Hanbin Mao* [1,2,5 *]

[1] Department of Chemistry and Biochemistry, Kent State University, Kent, OH 44240, USA

[2] School of Biomedical Sciences, Kent State University, Kent, OH 44240, USA

[3] Twinsburg High School, OH 44087, USA

[4] Department of Biochemistry and Molecular Biology, The College of Wooster, OH 44691, USA

[5] Advanced Materials and Liquid Crystals Institute, Kent State University, Kent, OH 44240, USA

[†] These authors contributed equally.

[*] Corresponding author: Hanbin Mao (hmao@kent.edu)

Contents

S1. List of oligonucleotides 3

S2. Calculation of effective ultrasound power 4

S3. Rolling circle amplification (RCA) 6

S4. Optical tweezers experiment 9

S5. Change-in-contour length (ΔL) histogram and theoretical ΔL 12

S6. Gel analysis of RCA DNA with and without ultrasound 13

S7. Ultrasound-mediated unfolding of single-unit hairpins 14

S8. Effect of template length on ultrasound-mediated unfolding 15

S9. Assessment of cell viability using the MTT assay 16

S10. Cellular uptake of doxorubicin and doxorubicin-loaded RCA 17

S11. Flow cytometry 18

S12. Titration of doxorubicin against hairpin-RCA 19

S13. Calculation of doxorubicin loading into AS1411-hairpin RCA 20

S14. Circular dichroism of AS1411 G-quadruplex 21

S15. Single-cell fluorescence imaging of doxorubicin treated cells 22

S16. Calculation of % hairpin unfolding 23

S17. Change in fluorescence during and after ultrasound 24

S18. Ultrasound-mediated unfolding in the presence of DMSO 25

S19. Staining cells with DAPI 26

S20. References 27

S1. List of oligonucleotides

Oligo ID	Length (nts)	Sequence (5’-3’)
single-unit hairpin	32	AAC CTA CTA CCT CAT TTT TGA GGT AGT AGG TT
Hairpin RCA template	56	GTC GTG ATA ACC TAC TAC CTC AAA AAT GAG GTA GTA GGT TGT ATA GTT CAA TCC TG
Random ssDNA RCA template	53	GTC GTG ATG TAC CAT TCA GAA CTG TCC ACC ATC TCC ACA AAG CTT CAA TCC TG
AS1411 RCA template	62	GTC GTG ATG TAC CAT TCA GAA CTG AAC CAC CAC CAC CAC AAC CAC CAC CAC CAA CAA TCC TG
single-unit AS1411	86	CGG TCA AAT ACT GTC CTT CTA GTG TAG CTT TGG TGG TGG TGG TTG TGG TGG TGG TGG TTT GCC AGC AAG ACG TAG CCC AGC GCG TC
splint	42	GCA TTA GGA AGC AGC CCA GTA GTA GGA TCA CGA CCA GGA TTG
splint remover	42	CAA TCC TGG TCG TGA TCC TAC TAC TGG GCT GCT TCC TAA TGC
AS1411-HP RCA 1.0	24	GGA CCC TGA AAA CAG GGT CCG GAC
AS1411-HP RCA 2.0	48	CGC TAG ACT GAA GTT TTT TTT TTT TTT TTT TTT TCT ACT ACC TCA CAG
AS1411-HP RCA 3.0	48	GAG GTA GTA GTT TTT TTT TTT TTT TTT TTT TCT TCA GTC TAG CGG TCC
AS1411-HP RCA 4.0	54	ATC GAC CGT AAA CCA CCA CCA CCA CAA CCA CCA CCA CCA AAA CGG TCG ATC TGT
$(dA)_{21}$	21	AAA AAA AAA AAA AAA AAA AAA
toehold 1	24	GCT ACA CTA GAA GGA CAG TAT TTG
toehold 2	30	CAG GGA CGC GCT GGG CTA CGT CTT GCT GGC
Telomeric GQ	27	TTA GGG TTA GGG TTA GGG TTA GGG TTA

S2. Calculation of effective ultrasound power

We used a custom hydrophone to measure ultrasonic power experienced by the sample. The hydrophone consisted of a sourced piezoelectric ceramic discs (Cheerock, Amazon) composed of lead zirconate titanate. Since these hydrophones had high sensitivity leaving unmodified discs susceptible to detector saturation, a synthetic flexible rubber polymer coating was used to dampen the high acoustic pressure fields created from ultrasonic applications. This allowed detected signals to be within an acceptable dynamic range for waveform analysis.

Acoustic signals from the hydrophone were recorded using a digital oscilloscope (Hantek 6022BE, 20 MHz bandwidth, 48 MSa/s sampling rate). The oscilloscope enabled waveform data capture in 30s intervals at a fixed sampling rate of 100 kHz, providing sufficient resolution to characterize the ultrasonic signals experienced by the sample. The voltage data was used to calculate RMS voltage values, which were subsequently used to derive acoustic pressure and intensity.

The RMS voltage is a statistical measure of average amplitude of an oscillating voltage signal over a representative time.

$$V_{RMS} = \sqrt{\frac{1}{N}\sum_{i=1}^{N} V_i^2}$$

With a known sensitivity of a hydrophone (M, in V/Pa) the acoustic pressure (P) can be calculated using:

$$P(Pa) = \frac{RMS(V)}{M(\frac{V}{Pa})}$$

Since all measurements were performed in water, a medium with known acoustic properties, acoustic intensity (ultrasound power) (I) can be calculated using:

$$I = \frac{Pa^2}{2\,pc}$$

I = Acoustic intensity or ultrasound power (W/m^2)
Pa = Pressure Amplitude
p = density of the medium (kg/m^3)
c = speed of sound in medium (m/s)

To ensure quantitative accuracy of the custom hydrophone, they were calibrated against a commercial reference hydrophone (model H1a from Aquarian Audio) under identical experimental setups. Following IEC 60565-1:2020, the response of each hydrophone under stable acoustic fields using ultrasonic transducers was recorded. By comparing the RMS of the custom to the reference hydrophone, the sensitivity of the custom hydrophone was found.

The peak power of ultrasound experienced by the sample from Branson 450 digital sonicator was found to be 5.3 mW/cm^2 and that of sonication bath FS30 sonic cleaner (Fisher Scientific) was found to be 0.46 W/cm^2.

To calculate the effective ultrasound power (P_{avg}) for a pulsed ultrasound, we used,

$$P_{avg} = P_{peak} \times DC,$$

where, P_{peak} is the peak power of ultrasound, DC is the duty cycle which is the fraction of time that the ultrasound is actively transmitted within one cycle.

The duty cycles used to achieve different sonication power in this study are given below:

Sonication power (mW/cm^2)	ON duration (sec)	OFF duration (sec)
0	0	-
2.0	5	10
3.7	15	10
4.6	30	10
5.0	45	10
5.3	60	10

S3. Rolling circle amplification (RCA)

Schematics of RCA[1,2] preparations are shown in Figures S1-S4. To prepare an RCA template, a linear ssDNA template consisting of a sequence complementary to the DNA of interest (i.e., hairpin or AS1411) was first phosphorylated at 37 °C for 3 hours using T4 PNK (NEB). The enzyme was heat-deactivated at 65 °C for 20 minutes. The phosphorylated template was then mixed with a *splint* DNA in 1:4 ratio and heated at 95 °C for 5 minutes and slowly cooled to 25 °C at the rate of −1 °C/min to facilitate circularization of the templates. The *splint* consists of two regions complementary to the flanking sequences of the linear ssDNA template. During this process, the DNA *splint* hybridized to the linear template, making it circular. The circularized ssDNA template was then ligated at 16 °C for 16 hours using T4 DNA Ligase (NEB) to link the 3' and 5' ends with a phosphodiester bond. The enzyme was heat-deactivated at 65 °C for 20 minutes. It was then mixed with the *splint remover* whose sequence was exactly complementary to the splint. The removal started at 95 °C for 10 minutes, 55 °C for 10 minutes, and 25 °C for 10 minutes. At this point, the circularized templates became free from the splint. Finally, the reaction mixture was subjected to exonucleases I and III (NEB) at 37 °C for 12 hours to chop off any linear ssDNA/dsDNA. The exonuclease enzymes were then heat-deactivated at 80 °C for 20 minutes.

To prepare an ssDNA construct containing an array of DNA secondary structures (Figures S1-S3), the circularized template prepared above was hybridized with a primer (*splint*) in 3:1 mole ratio at 95 °C for 5 minutes followed by cooling to 25 °C at the rate of −1 °C/min. The primer annealed to the circular template and the rolling cycle amplification (RCA) was carried out by the Phi29 DNA polymerase (NEB) for 10 minutes at 37 °C. The polymerase enzyme was then heat-deactivated at 65 °C for 20 minutes resulting in the final RCA product. Finally, the RCA product was filtered using a 100K molecular cut-off Amicon® micro-centrifugal filters (Merck, USA).

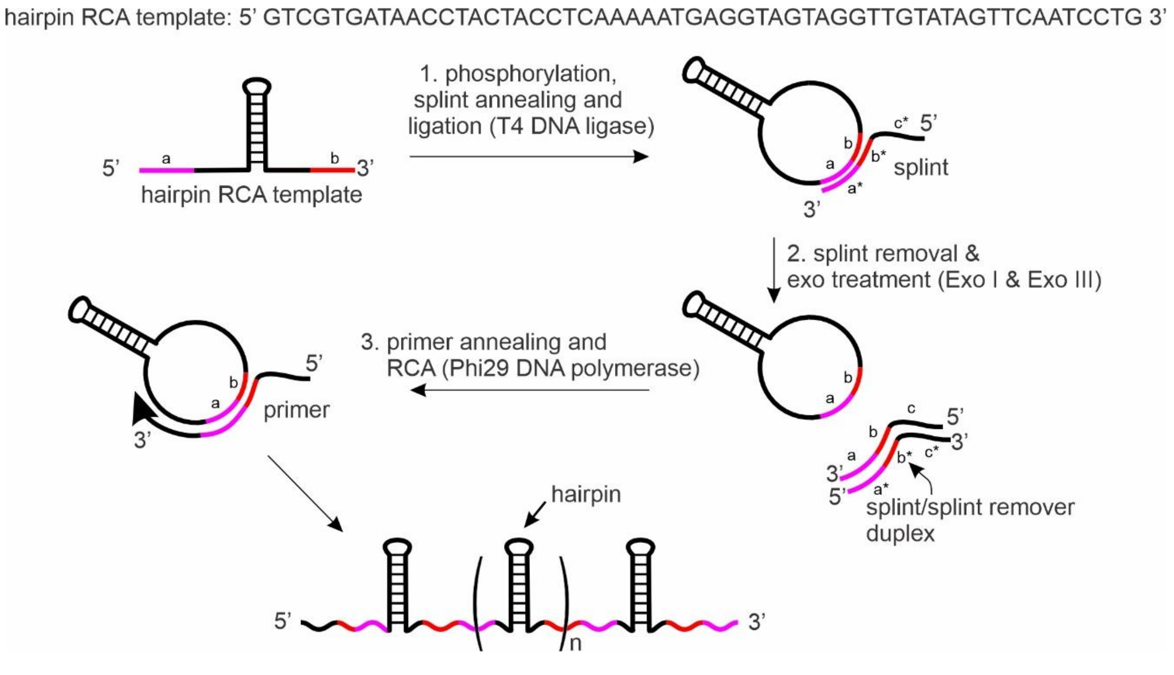

Figure S1. Schematic of rolling circle amplification (RCA) protocol for the synthesis of long ssDNA consisting of tandem array of hairpin structures.

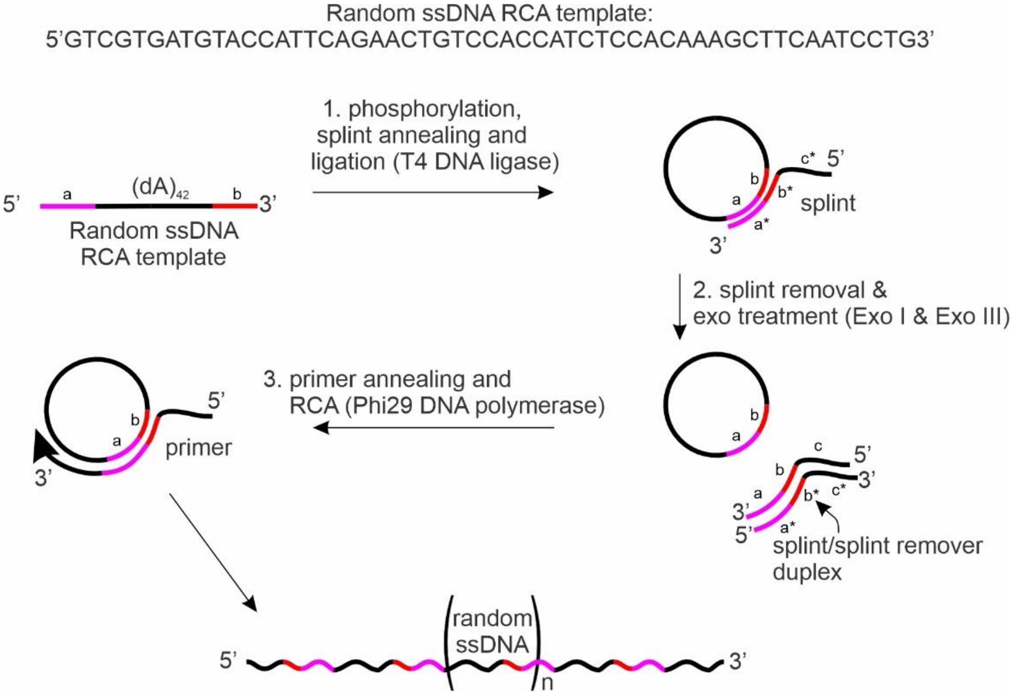

Figure S2. *Schematic of rolling circle amplification (RCA) protocol for the synthesis of long ssDNA consisting of tandem array of a random sequence.*

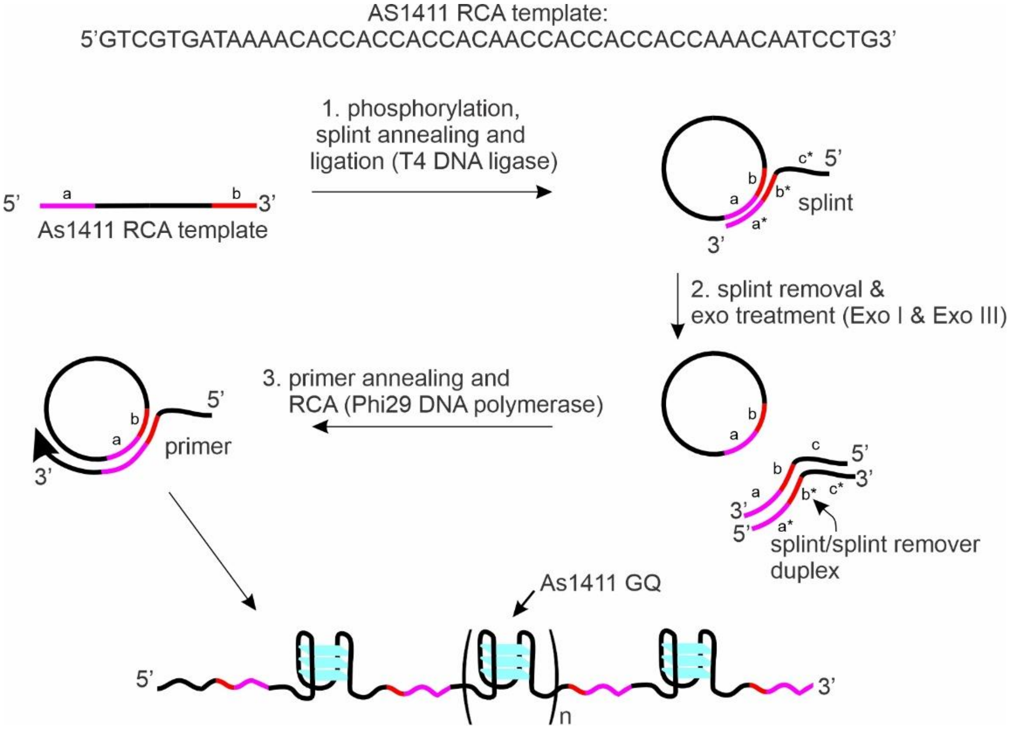

Figure S3. *Schematic of rolling circle amplification (RCA) protocol for the synthesis of long ssDNA consisting of tandem array of AS1411 G-quadruplex structures.*

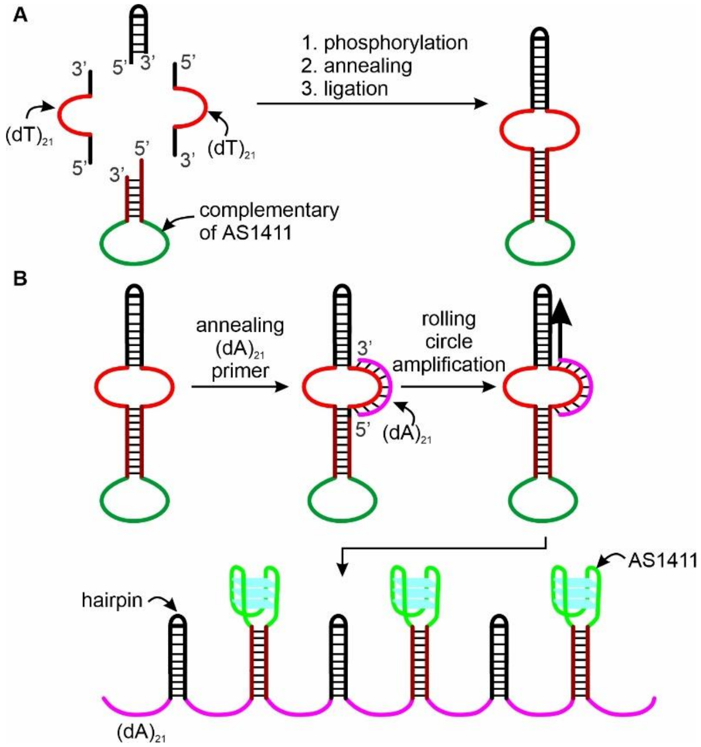

Figure S4. *Schematic of rolling circle amplification (RCA) protocol for the synthesis of a long ssDNA consisting of an array of AS1411-HP sequence.*

To prepare the template for the AS1411-hairpin (AS1411-HP) RCA construct (Figure S4), four oligos (AS1411-HP RCA 1.0/2.0/3.0/4.0) were first phosphorylated separately at 37 °C for 3 hours T4 PNK (NEB). The enzyme was heat-deactivated at 65 °C for 20 minutes. The phosphorylated oligos were then mixed together in 1:1:1:1 mole ratio and heated at 95 °C for 5 minutes and slowly cooled to 25 °C at the rate of −1 °C/min to facilitate annealing of the oligos. The annealed template was then ligated at 16 °C for 16 hours using T4 DNA Ligase (NEB) to link the 3' and 5' ends with a phosphodiester bond. The enzyme was heat-deactivated at 65 °C for 20 minutes.

To prepare a ssDNA construct containing an array of hairpin structures and AS1411 GQ, the ligated circular template prepared above was hybridized with a primer (dA_{21}) in 10:1 mole ratio at 95 °C for 5 minutes followed by cooling to 25 °C at the rate of −1 °C/min. The primer annealed to the template and the rolling cycle amplification (RCA) was carried out by the Phi29 DNA polymerase (NEB) for 15 minutes at 37 °C. The polymerase enzyme was then heat-deactivated at 65 °C for 20 minutes resulting in the final RCA product. Finally, the RCA product was filtered using a 100K molecular cut-off Amicon® micro-centrifugal filters (Merck, USA).

S4. Optical tweezers experiment

All the single-molecule nucleic acid templates were studied in a custom built dual-trap optical tweezers instrument[3]. The two laser traps were used to trap two polystyrene beads (diameters: 1.76 μm and 2.32 μm) coated with streptavidin and anti-digoxigenin antibody, respectively. Streptavidin and anti-digoxigenin antibody coated polystyrene beads were purchased from Spherotech Inc., USA and ready-to-use. A single-molecule nucleic acid template was tethered between the two beads via biotin-streptavidin and digoxigenin (dig)/anti-dig linkages (see Figures 1E and 2D, and Figures S6 and S7). A tension was applied to the tethered template by moving the two optically trapped beads apart using a steerable mirror that controls one of the trapping lasers. The tension was calculated based on the spring constant of each trap and the displacement of the beads from the center of the trap. The force *vs* extension (FX) profile of the templates was recorded using a LabView program (National Instruments, Austin, TX) at 1 kHz frequency and a loading rate of 5.5 pN/sec (10-30 pN range). Custom-written MATLAB (The MathWorks) scripts were used to acquire and process those data. The MATLAB processed data were then analyzed using IGOR Pro v6.37 (WaveMetrics) software. The custom-written IGOR programs were used to plot the Force-Extension (FX) curves.

To study the unfolding of hairpin and AS1411 GQ structures, a three-channel microfluidic chamber was used as shown in Figure S5 below. In brief, the streptavidin-coated bead conjugated with the nucleic acid template and the anti-dig coated beads flowed respectively from the bottom and top channels to the middle channel *via* microcapillary tubes. Two different beads were then captured separately in the middle channel by the two laser traps in 10 mM Tris buffer (pH 7.4) supplemented with 100 mM KCl. To study the unfolding force of those structures, the single-molecule nucleic acid template was stretched and relaxed repeatedly while recording the Force-Extension profile simultaneously.

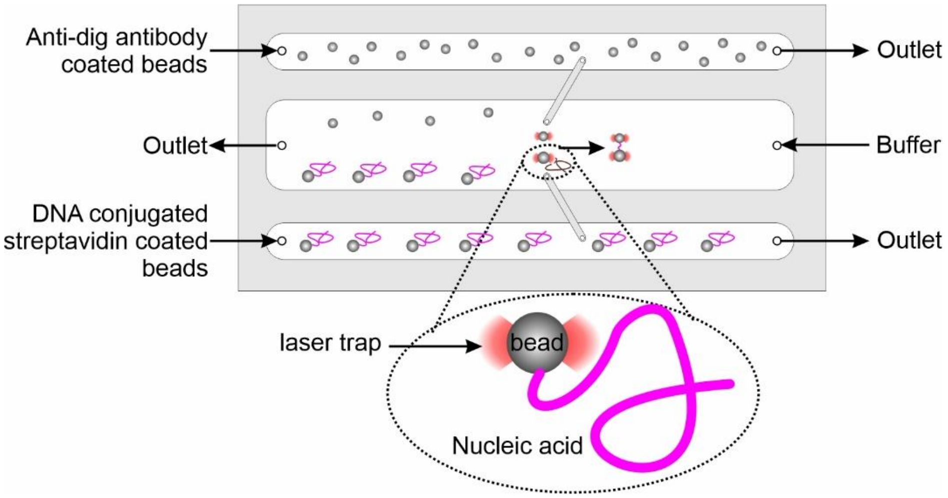

Figure S5. *Schematic of a microfluidic chamber to perform single-molecule force spectroscopy on the RCA products.*

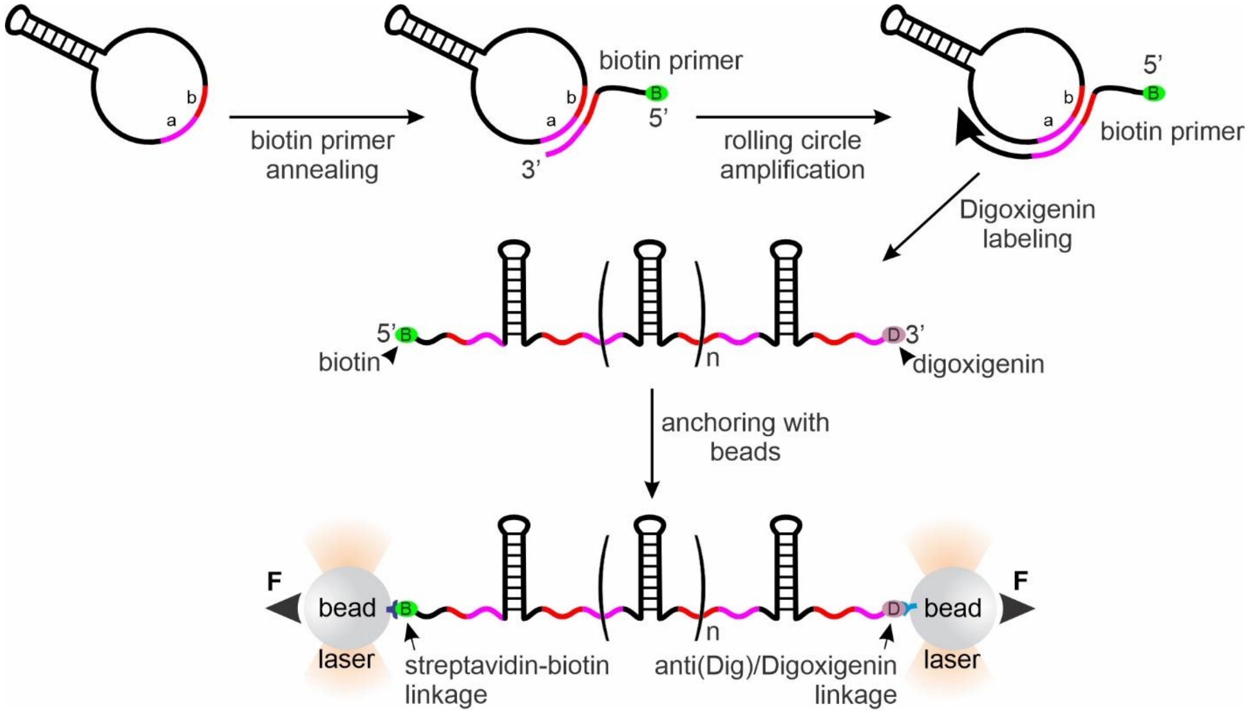

Figure S6. *Schematic of synthesis of hairpin RCA for optical tweezers experiment.*

Following the synthesis of a circular template (see SI section S3 above), a biotinylated primer (5’-biotin-*splint*) was annealed to the circular template by heating their mixture (1:3 mole ratio) at 95 °C for 5 minutes followed by cooling to 25 °C at the rate of −1 °C/min. The primer annealed to the circular template and RCA was carried out at 30 °C for 10 minutes using Phi29 DNA polymerase (NEB). The 3’ end of the RCA product was modified with digoxigenin(dig)-dUTP (Enzo life sciences) using Terminal deoxynucleotidyl Transferase (TdT, Thermofisher) at 37 °C for 2 hours. The enzyme was deactivated by heating at 75 °C for 20 minutes. Now, the final RCA-based ssDNA construct had 5'-biotin and 3'-digoxigenin labeled, which facilitated the tethering between the two trapped beads in the optical tweezers instrument.

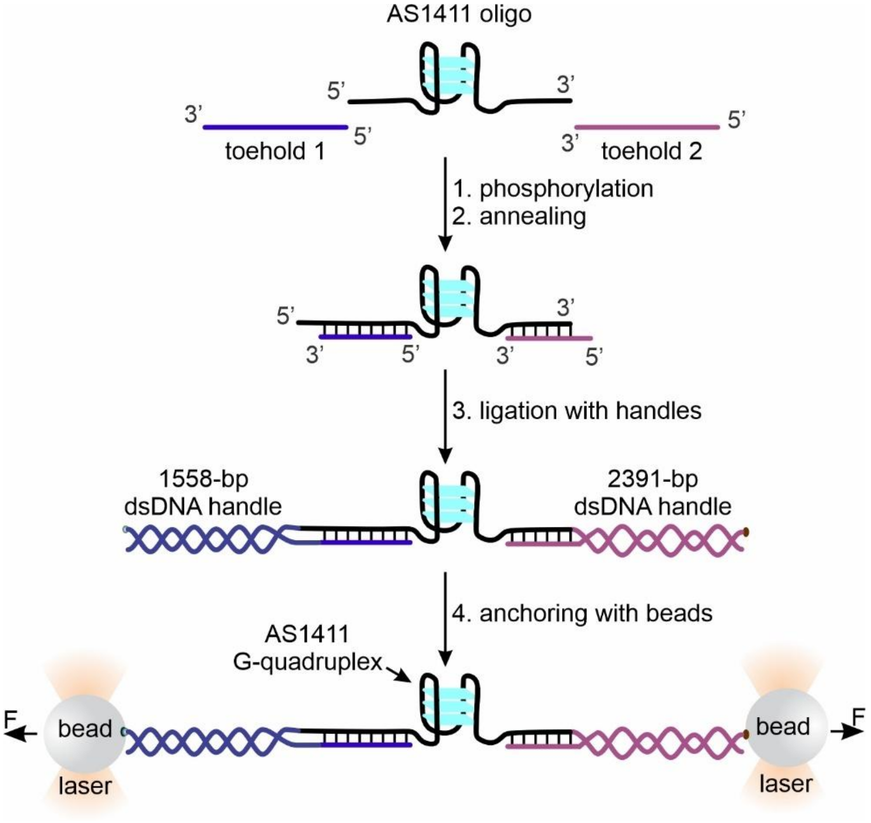

Figure S7. *Synthesis of a single molecule AS1411 GQ construct for optical tweezers experiment.*

First, a *single-unit AS1411* sequence (see sequence in SI Table S1) and *toehold 2* were separately phosphorylated at 37 °C for 3 hours using T4 PNK (NEB). T4-PNK was deactivated by heating the reaction mixture at 65 °C for 20 minutes. Phosphorylated *single-unit AS1411, toehold 1, and phosphorylated toehold 2* were then annealed together by heating their mixture (1:1:1 mole ratio) at 95 °C for 5 minutes followed by cooling to 25 °C at the rate of −1 °C/min. The annealed product was then ligated with 2391 bp dsDNA and 1558 bp dsDNA handles (3:1:1 mole ratio) at 16 °C for 16 hours using T4 DNA Ligase (NEB). 2391 bp dsDNA handles contained digoxigenin on one end and 1558 bp dsDNA handles contained biotin on one end facilitated the tethering between the two optically trapped beads in the optical tweezers instrument. Preparation of the 2391 bp and 1558 bp dsDNA handles was reported in our previous paper[4,5].

S5. Change-in-contour length (ΔL) histogram and theoretical ΔL

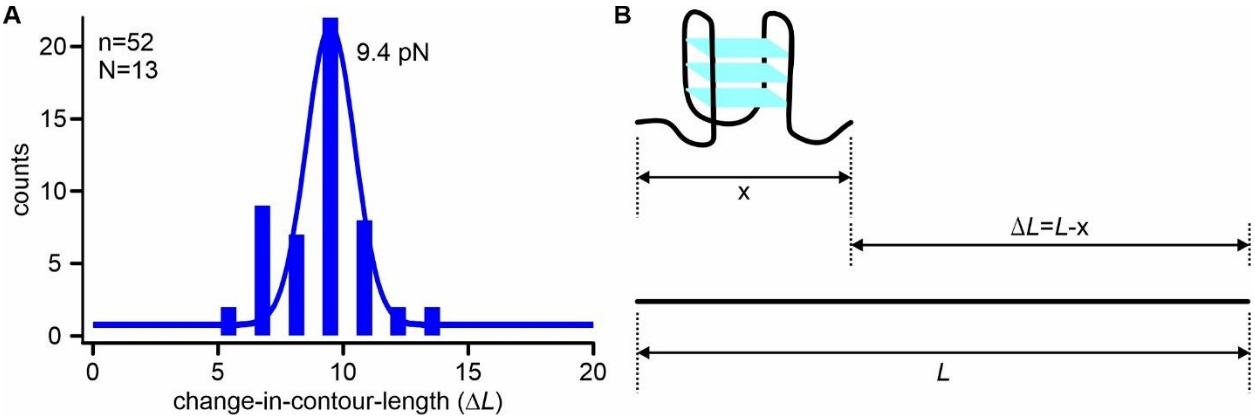

Figure S8. *(A) Change-in-contour-length (ΔL) histogram during mechanical unfolding of the AS1411 GQ construct. (B) Calculation of theoretical ΔL.*

To calculate the theoretical ΔL,

L= length of fully unfolded AS1411 structure = total no. of nucleotides × length of a nucleotide

= 26 × 0.45 nm[6,7]

= 11.7 nm

x= end-to-end length of folded AS1411 structure = 2.5 nm[8]

change-in-contour-length (ΔL) = L-x

= 11.7 – 2.5 nm

= 9.2 nm

S6. Gel analysis of RCA DNA with and without ultrasound

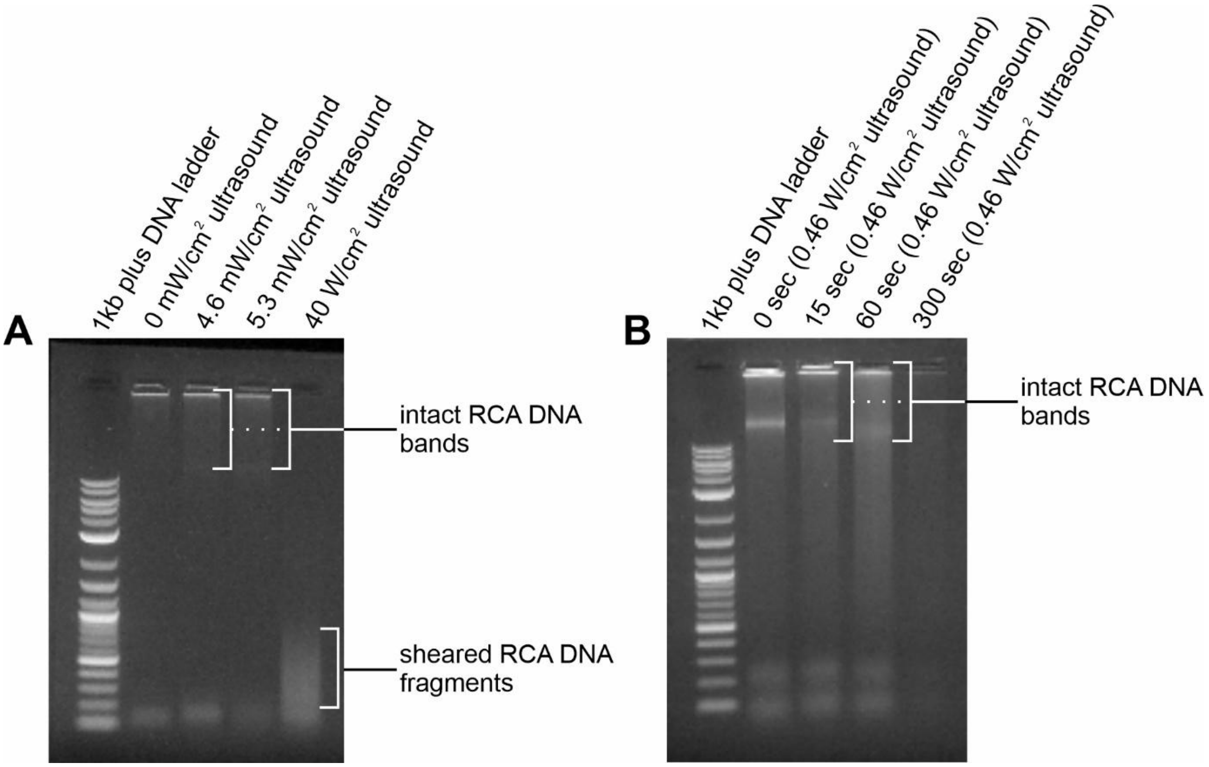

Figure S9. *Gel analysis of (A) hairpin RCA construct without and with 15 minutes of sonication at different powers. The DNA construct remained intact with negligible damage when the sonication power was maintained below 5.3 mW/cm^2. However, when the power was significantly high (40 W/cm^2) the hairpin RCA DNA was heavily sheared leaving fragments less than 2000 nt in length. (B) Gel analysis of AS1411-HP RCA construct with 0.46 W/cm^2 sonication for different times. The DNA construct remained intact with negligible damage when the sonication time was less than a minute. However, when it was sonicated for 5 minutes, a considerable damage was observed.*

S7. Ultrasound-mediated unfolding of single-unit hairpins

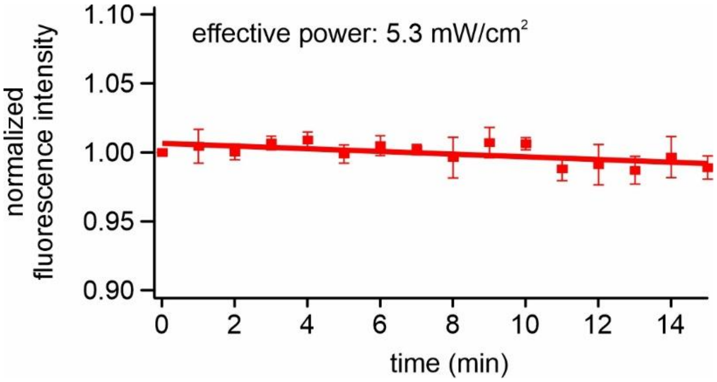

Figure S10. *Unfolding of single-unit hairpin (see SI Table S1 for sequence) using probe based ultrasonication (Digital Sonifier 450, Branson Ultrasonics Corporation) as described in Figure 1. The fluorescence intensity didn't change for as long as 15 minutes even at the highest ultrasound power (5.3 mW/cm^2).*

S8. Effect of template length on ultrasound-mediated unfolding

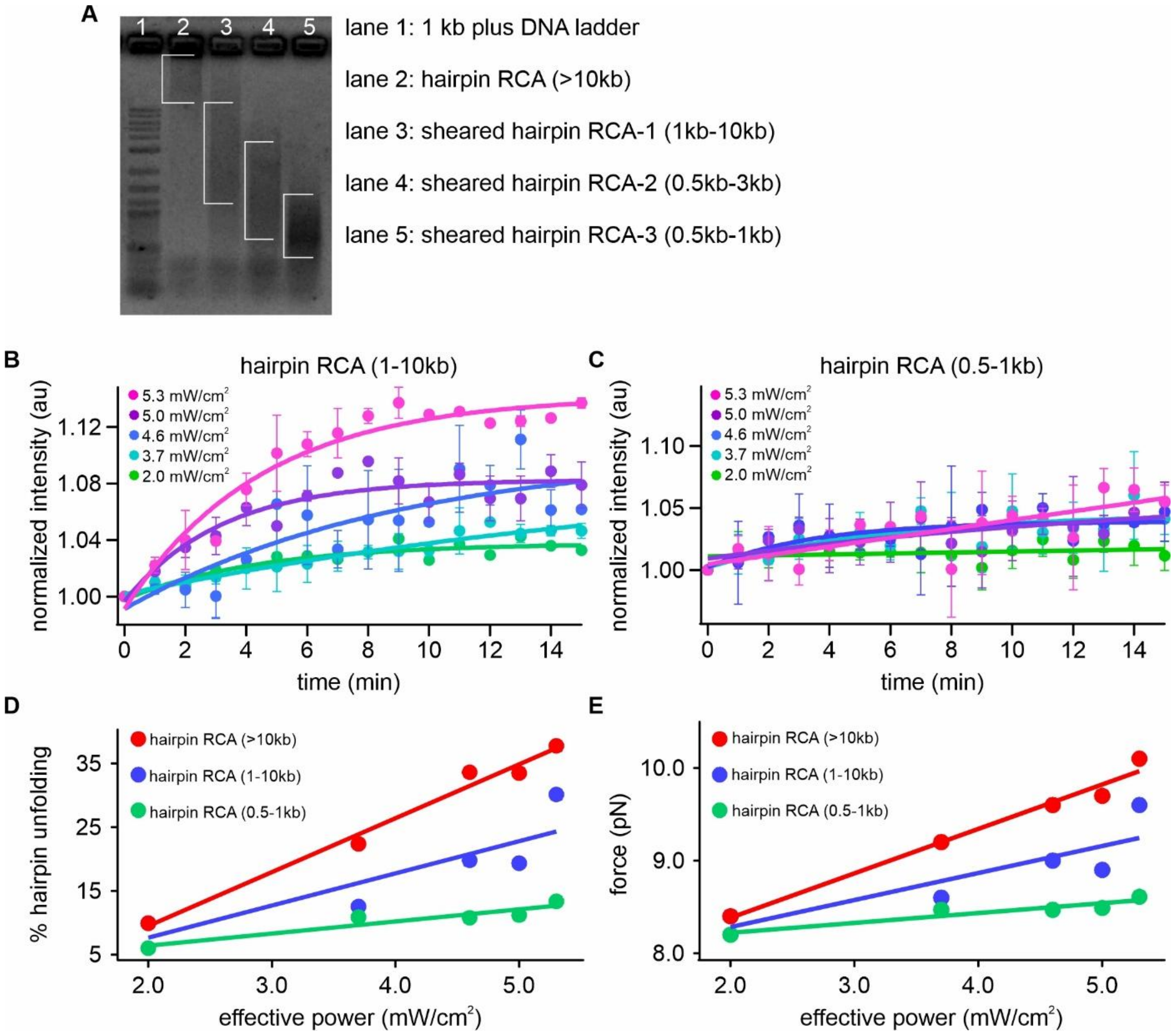

Figure S11. *Effect of the length of RCA constructs on ultrasound-mediated unfolding of hairpin structures within the RCA constructs. (A) Gel image of RCA products with different lengths. (B) ultrasound-mediated unfolding of hairpin RCA-1 (1kb-10kb). (C) ultrasound-mediated unfolding of hairpin RCA-3 (0.5kb-1kb). (D) % hairpin unfolding at different ultrasound power for hairpin RCA constructs with different lengths. (E) Force calibration of hairpin RCA constructs with different lengths.*

S9. Assessment of cell viability using the MTT assay

HeLa cells obtained from ATCC were cultured in DMEM/F12 50:50 (DMEM/F12; Gibco, NY, USA) supplemented with 10% (*v/v*) fetal bovine serum (FBS, Gibco) and 1% penicillin/streptomycin (Gibco) until 90% confluent (2-4 days). Cells were incubated at a temperature of 37 °C with a minimum relative humidity of 95%, and an atmosphere of 5% CO_2.

To assess the cell viability in the presence of doxorubicin, AS1411-hairpin RCA, or the mixture of doxorubicin and AS1411-hairpin RCA, MTT (3-(4,5-dimethylthiazol-2-yl) 2,5-diphenyl-tetrazolium bromide) assay was used. First, the cells were plated in 96 well culture plates at a density of 20×10^3 cells/well and incubated in CO_2 incubator at 37 °C temperature, 95% relative humidity, and 5% CO_2. After 24 hours, cells were treated with the varying concentration of the agents (Doxorubicin, AS1411-hairpin RCA, or doxorubicin loaded AS1411-hairpin RCA) and incubated for another 24 hours under same condition. Then, before MTT assay, the cell medium (serum containing) was aspirated and a fresh serum free media containing MTT reagent (0.5 mg/mL) was added to each well, and the cells were again incubated at 37 °C. The live cells that are metabolically active take up MTT and reduce it into insoluble purple formazan crystals with the help of mitochondrial dehydrogenase After 3 hours, the serum free medium was removed by aspiration, and formazan crystals formed by the viable cells were dissolved using the same volume (100 µL) of DMSO. The plates were shaken for 5 minutes on a plate shaker to ensure adequate solubility of formazan crystals. Then the plate was read at 562 nm by using the microtiter plate reader (Biotek elx808). The results were analyzed as the percentage of live cells in respect to the concentration of the samples treated.

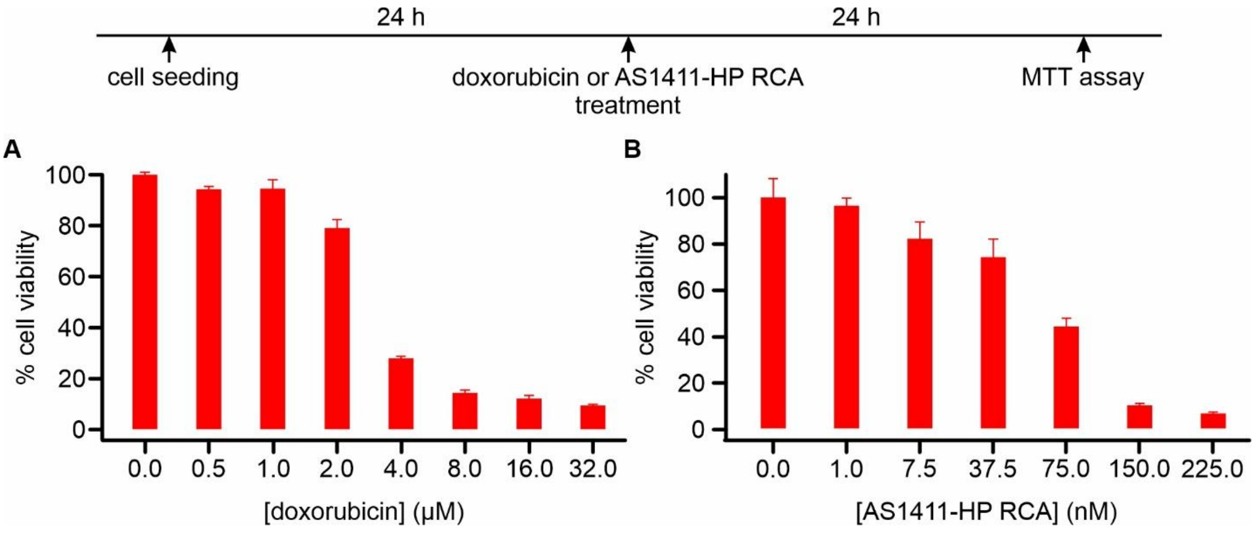

Figure S12. *Viability of HeLa cells against doxorubicin (A), and AS1411-hairpin RCA (B).*

S10. Cellular uptake of doxorubicin and doxorubicin-loaded RCA

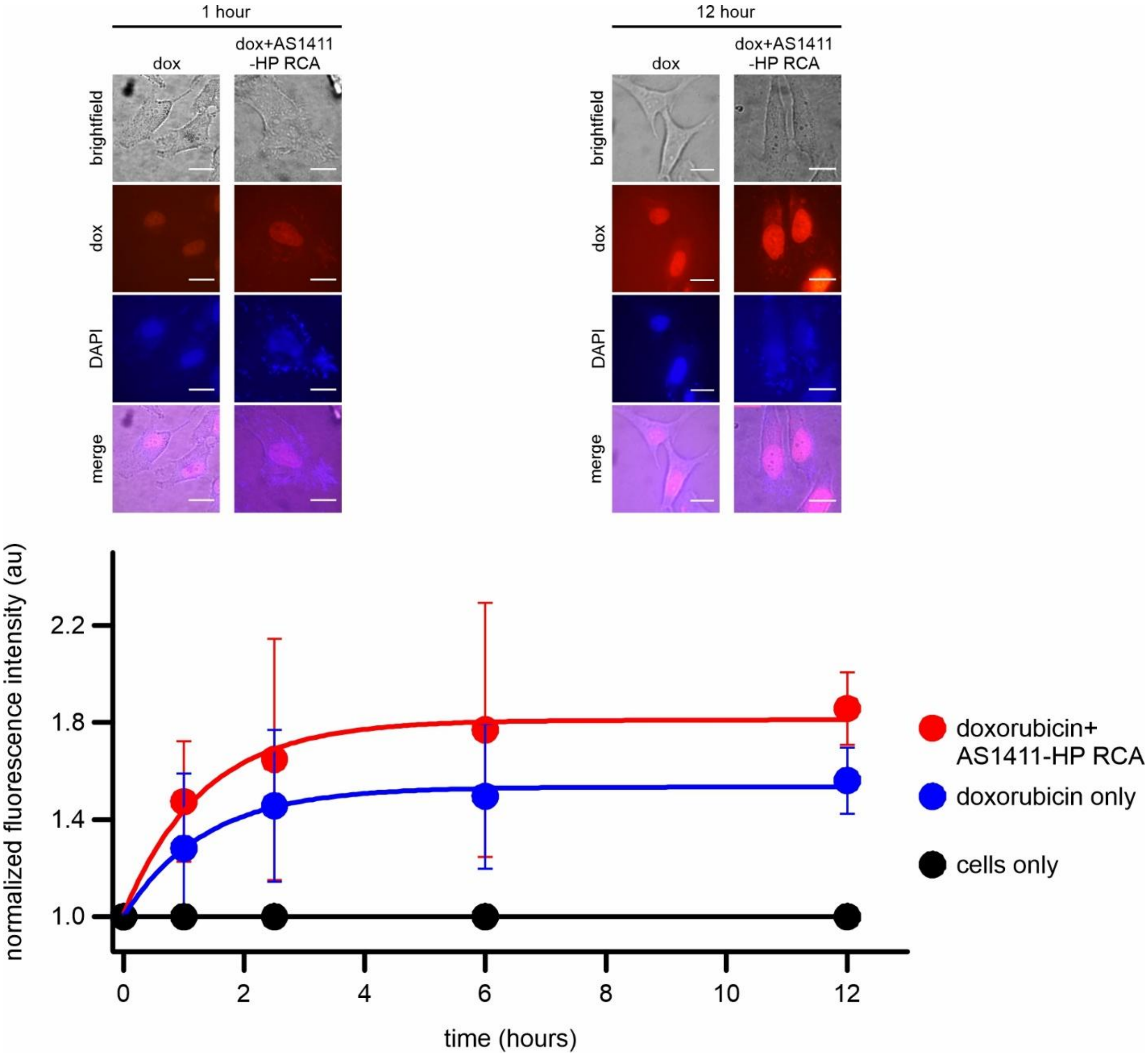

Figure S13. *Single-cell fluorescence imaging of cells loaded with 2 µM doxorubicin only (blue), 2µM doxorubicin-loaded 37.5 nM AS1411-HP RCA (red), and untreated cells (black). Scale bar: 5 µm. Two tailed student t-tests reveal no significant difference ($p > 0.05$) in each data point between 1 and 12 hours.*

HeLa cells were seeded in an image dish (VWR) at a density of 20k cells/dish and were incubated overnight at 37 °C to allow attachment. After 24 hours, cells were treated with 2 µM doxorubicin and 2 µM doxorubicin loaded 37.5 nM AS1411-HP RCA. After treatment, the cells were imaged for fluorescence under a confocal fluorescence microscope (Nikon Eclipse TE2000-U Inverted Microscope) at different time intervals. Each data point represents an average fluorescence intensity of at least 20 individual cells.

S11. Flow cytometry

HeLa cells were seeded on a 6-well plate at a density of 500k cells/well and were incubated overnight at 37 °C to allow attachment. After 12 hours, cells were treated with 2 μM doxorubicin and 2 μM doxorubicin loaded 37.5 nM AS1411-hairpin RCA. A control group of cells with treatments were not subjected to ultrasound, whereas another group of cells with treatments were subjected to the same ultrasound in a sonication bath to investigate the effect of ultrasound on the intracellular release of doxorubicin. After 1 hour of treatment, the cells in the control group were harvested by trypsinization. To another group, ultrasound was applied for 15 seconds in the sonication bath at effective power of 0.46 W/cm^2 followed by cells harvesting. The harvested cells were centrifuged, and the cell pellets were washed with PBS and again resuspended in PBS pH 7.4. Data were acquired to determine the proportion of viable cells, and cells with doxorubicin fluorescence.

NovoCyte Flow Cytometer (Agilent Technologies) instrument equipped with NovoSample Pro automatic sampler (Agilent Technologies) was used for the flow cytometry experiment, and after data collection, results had been analyzed using the in-program functions and statistical comparisons had been made.

S12. Titration of doxorubicin against hairpin-RCA

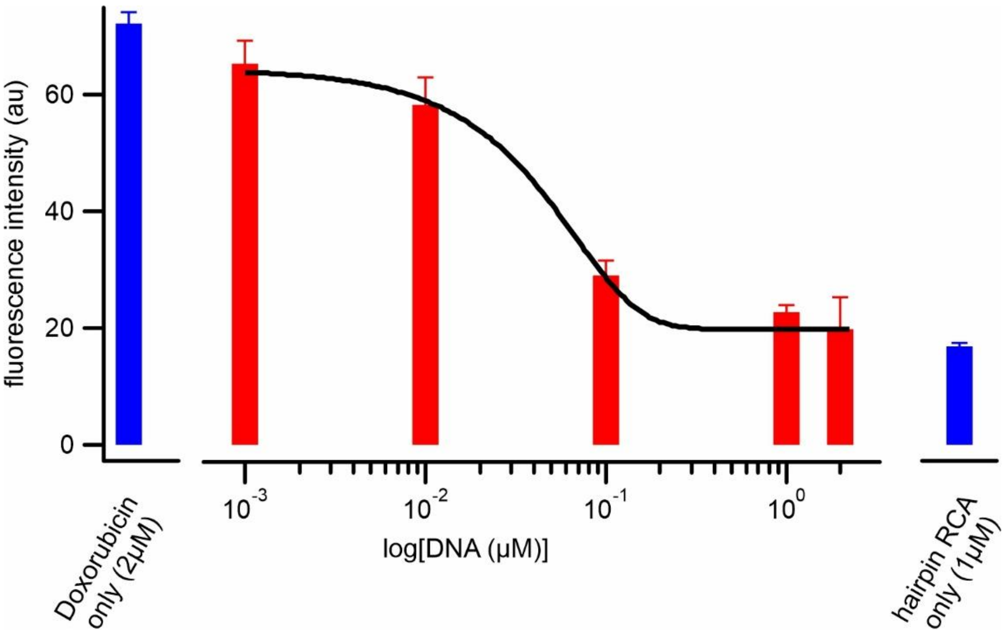

Figure S14. *Titration of doxorubicin against hairpin RCA to determine the working concentration used in experiments. Doxorubicin was fixed at 2 μM concentration and was mixed with different concentrations of hairpin RCA. After incubating for 10 minutes, fluorescence intensity was measured under a confocal fluorescence microscope (Nikon Eclipse TE2000-U Inverted Microscope). Black fitted curve represents sigmoidal fitting. Blue bar represents the boundary of the fluorescence intensity when there is only doxorubicin (no hairpin RCA) and only hairpin RCA (no doxorubicin). From this experiment, we chose to use 1 μM hairpin RCA construct to mix with 2 μM doxorubicin in the experiments described in Figure 1, as it had the lowest background compared to higher concentrations, indicating that doxorubicin fluorescence was close to fully quenched.*

S13. Calculation of doxorubicin loading into AS1411-hairpin RCA

Doxorubicin intercalates into double-stranded DNA (dsDNA) and typically occupies two to three base pairs upon binding[9]. The exact number can vary depending on the DNA sequence and experimental conditions, but most studies suggest it intercalates between two base pairs, causing local unwinding and structural distortion of the DNA helix.

Length of duplex stem in a single repeat in the AS1411-hairpin RCA construct = 50 base pairs.

Doxorubicin occupancy = 2 base pairs per doxorubicin molecule.[9]

Therefore, doxorubicin occupancy in a single repeat in the AS1411-hairpin RCA construct= 50/2 base pairs = 25 base pairs, which gives the ratio of doxorubicin to single repeat in the AS1411-hairpin RCA construct as 25:1.

Experimental concentrations of doxorubicin and RCA are 2 μM and 37.5 nM, respectively, which gives the ratio of 53:1. This indicates that RCA construct is fully loaded with doxorubicin molecules.

S14. Circular dichroism of AS1411 G-quadruplex

To confirm whether G-quadruplex (GQ) structure is formed in the AS1411 sequence or in the AS1411-RCA constructs, we performed circular dichroism (CD) experiment. CD spectra were recorded on Jasco J-810 spectropolarimeter (Jasco, Easton, MD). A quartz cell of 1 mm optical path length and an instrument scanning speed of 100 nm/min with 1 s response time was used for measurements. CD spectra were recorded with a spectral bandwidth of 1.0 nm and a data pitch of 1.0 nm.

We measured CD spectra of 5 µM AS1411 RCA constructs in 10 mM Tris buffer (pH 7.4) supplemented with 100 mM KCl. We first prepared dNTP free AS1411-RCA products by filtration using 100k molecular cut-off Amicon® filter (Millipore Sigma, USA). It was then diluted in the buffer with a final concentration of 5 µM (concentration of the AS1411-RCA constructs was determined by NanoDrop One microvolume UV/Vis spectrophotometer, Fisher Scientific). The sample was heated at 95 °C for 10 minutes, followed by rapid cooling on an ice-bath for 30 mins. Finally, the CD spectra were collected in triplicates with their average plotted after smoothing using Savitsky-Golay function.

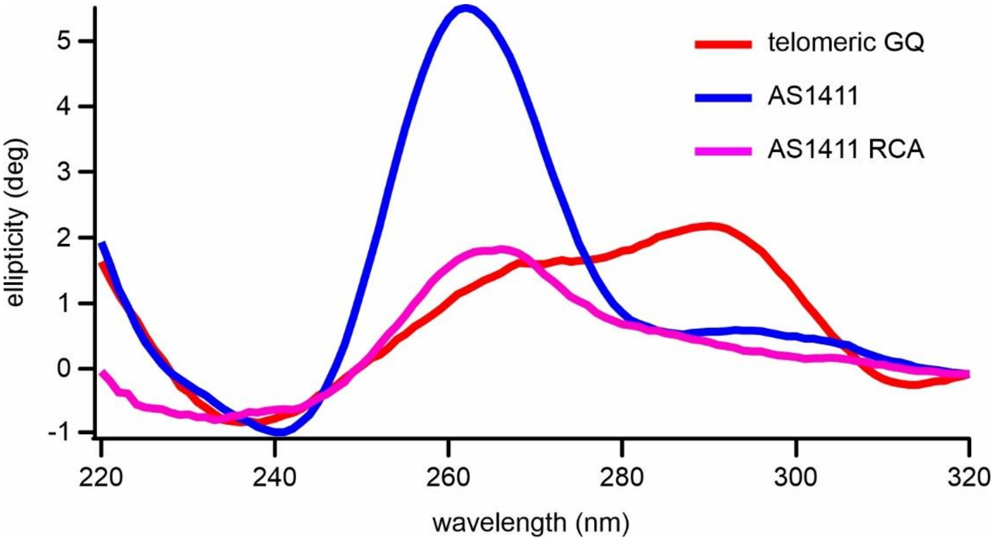

Figure S15. *CD spectra of telomeric GQ sequence (red), AS1411 sequence (blue), and AS1411 RCA construct (pink). See Table S1 for sequences.*

Telomeric G-quadruplex (telomeric GQ) is a commonly known GQ forming sequence[10]. The crests at ~295 nm and ~260 nm and a trough at ~240 nm in the CD spectra (red) suggest a hybrid GQ conformation[11]. The formation of a GQ structure in AS1411 sequence is confirmed by the characteristic crest at ~260 nm and trough at ~240 nm (blue) ,which suggest a parallel GQ conformation[8]. Compared to the CD spectra of the AS1411 sequence, the CD spectra of the AS1411 RCA construct (pink) also showed similar crest and trough positions characteristic of parallel GQ conformations. The reduced magnitude of the ~260 nm crest in the AS1411-RCA construct with respect to the AS1411 sequence is likely due to the random spacer sequence between two AS1411 GQ forming repeats in the AS1411-RCA construct (see SI Figure S3).

S15. Single-cell fluorescence imaging of doxorubicin treated cells

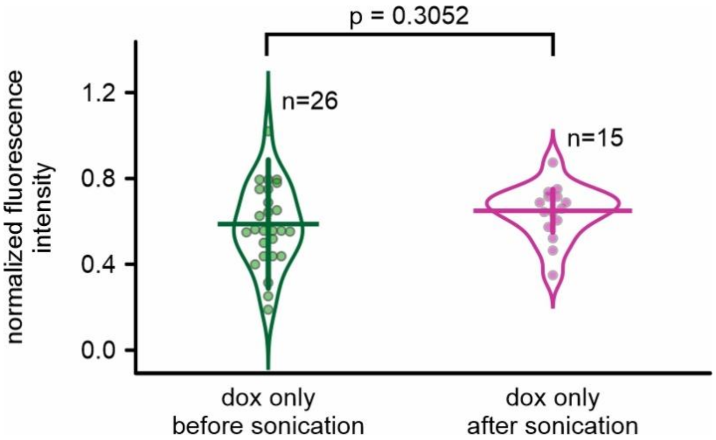

Figure S16. *Violin plots of fluorescence intensity obtained from single-cell fluorescence imaging of 2 μM doxorubicin treated HeLa cells before and after 15 seconds, 0.46 W/cm^2 sonication. Horizontal lines represent mean fluorescence intensities and vertical lines represent standard deviations. The numbers of cells measured are indicated by "n" in the figure.*

HeLa cells were seeded in an image dish (VWR) at a density of 20k cells/dish and were incubated overnight at 37 °C to allow attachment. After 24 hours, cells were treated with 2 μM doxorubicin and 2 μM doxorubicin loaded 37.5 nM AS1411-HP RCA. After 1 hour of treatment, the cells were imaged for fluorescence (green data) under a confocal fluorescence microscope (Nikon Eclipse TE2000-U Inverted Microscope). These cells were then sonicated for 15 sec at 0.46 W/cm^2 before being imaged again under the same microscope (purple data). There was no significant difference (p = 0.3052) in the fluorescence intensities between the cells treated with doxorubicin before and after sonication.

S16. Calculation of % hairpin unfolding

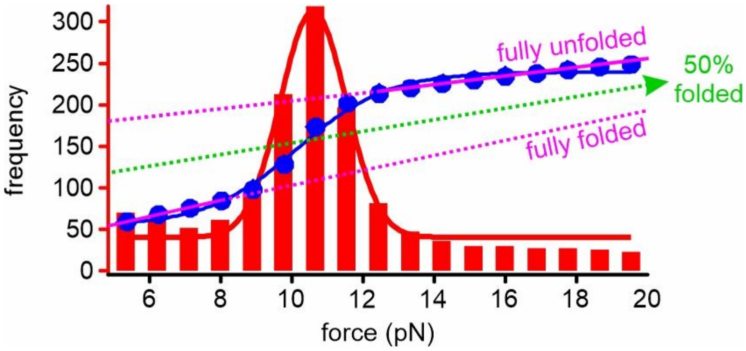

Figure S17. *Reconstructed force histogram (red) and cumulative unfolding percentage of hairpin structures in the RCA construct (blue). Solid red curve depicts Gaussian fitting, and two dotted pink lines represent the extrapolation depicting 100% and 0% folded hairpins, respectively. Green dotted line depicts 50% folded hairpins.*

First, a force histogram (red bars) was constructed from the force-extension curves obtained from the unfolding of hairpin RCA molecules. The population in the histogram represents the force at which hairpins formed within the RCA construct were unfolded. From the histogram, a cumulative unfolding percentage (blue solid dots) of hairpin was drawn. The plateau regions on both sides of the sigmoidal fitting (blue solid curve) represent the two boundaries of hairpin unfolding force. The lower plateau region suggests that the force is not enough to start unfolding the hairpin structures whereas the higher plateau region suggests that the force is high enough that all hairpin structures within the molecules are unfolded.

The two plateau regions were fitted with linear equations (pink dotted line). The lines were extrapolated, representing the boundaries of 0% and 100% hairpin unfolding, respectively, at a particular force. Using these boundary conditions, a straight line is plotted passing through the middle of the boundary conditions (green dotted line). The intersection of green dotted line and the sigmoidal fitting was considered as the force at which 50% of hairpins were unfolded.

S17. Change in fluorescence during and after ultrasound

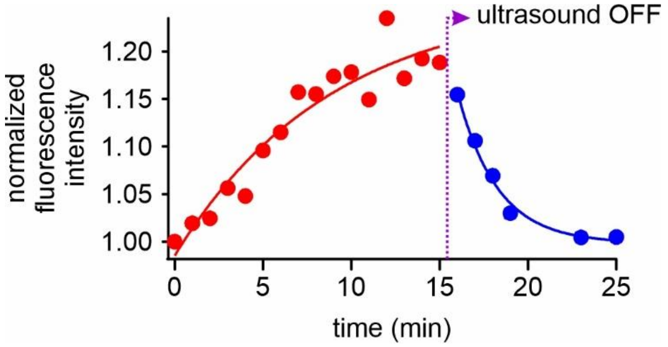

Figure S18. *Fluorescence quenching after sonication is stopped. 2 µM Doxorubicin-loaded hairpin RCA (1 µM) was sonicated using the probe based ultrasonication (Digital Sonifier 450, Branson Ultrasonics Corporation) for 15 minutes at 5.3 mW/cm*^{2}*. During sonication, the fluorescence intensity increased (red solid markers), indicating the unfolding of hairpin structures and the subsequent release of doxorubicin molecules. The solid red curve represents an exponential fit. Upon cessation of sonication after 15 minutes, the fluorescence intensity began to decline (blue solid markers), returning to its initial level within 5 minutes. The solid blue curve represents an exponential fit. This observation suggests that, in the absence of sonication, the hairpin structures refold, facilitating the rebinding of doxorubicin molecules and thereby reducing the fluorescence intensity.*

S18. Ultrasound-mediated unfolding in the presence of DMSO

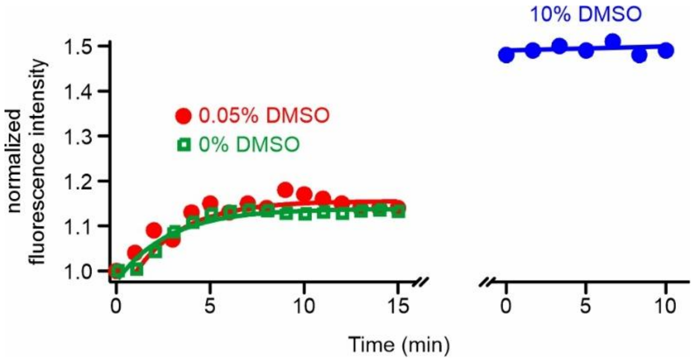

Figure S19. *Effect of DMSO on ultrasound-mediated unfolding of hairpin RCA (>10 kb). Hairpin RCA (1 µM), pre-loaded with 2 µM doxorubicin, was subjected to 5.3 mW/cm^2 sonication (Digital Sonifier 450, Branson Ultrasonics Corporation) for 15 minutes in a buffer containing 10 mM Tris, 100 mM KCl, pH 7.4. To assess the contribution of free radicals generated during sonication, 0.05% DMSO (red) was added as a radical scavenger. This low concentration of DMSO had negligible effect (red vs green) on the fluorescence change following the ultrasound treatment (solid curves depict exponential fittings with reaction rate constants of 2.8±0.5 and 2.6±0.6 min^{-1}, respectively, which shows no significant difference (p=0.3139) by student t-test), indicating that free radical production did not contribute to hairpin unfolding. Addition of 10% DMSO (blue) resulted in substantial destabilization of the hairpin structures[12], as indicated by elevated initial fluorescence, and therefore, no significant fluorescence increase was observed upon subsequent 5.3 mW/cm^2 sonication on the same sample. These results indicate that the fluorescence increase observed during sonication is primarily driven by mechanical shear forces rather than free radical-induced effects.*

S19. Staining cells with DAPI

Cultured untreated cells or those treated with 2 μM doxorubicin or 2 μM doxorubicin loaded AS1411-HP RCA were first washed with PBS 1-2 times to remove treatment agents. A 100 μL of 0.3 mM DAPI (4′,6-diamidino-2-phenylindole) (ApexBio Technology, USA) stain solution prepared in the DMEM/F12 50:50 (DMEM/F12; Gibco, NY, USA) media supplemented with 10% (v/v) fetal bovine serum (FBS, Gibco) and 1% penicillin/streptomycin (Gibco) was added. Cells were then incubated for 5 minutes at a temperature of 37 °C with a minimum relative humidity of 95%, and an atmosphere of CO_2. The stain solution was then removed and washed with fresh media before imaging. Finally, cells were imaged under a fluorescence microscope using DAPI channel with 350-380 nm excitation and 420-480 nm emission.

S20. References

(1) Liu, D.; Daubendiek, S. L.; Zillman, M. A.; Ryan, K.; Kool, E. T. Rolling Circle DNA Synthesis: Small Circular Oligonucleotides as Efficient Templates for DNA Polymerases. *J. Am. Chem. Soc.* **1996**, *118*, 1587.

(2) Ali, M. M.; Li, F.; Zhang, Z.; Zhang, K.; Kang, D.-K.; Ankrum, J. A.; Le, X. C.; Zhao, W. Rolling circle amplification: a versatile tool for chemical biology, materials science and medicine. *Chemical Society Reviews* **2014**, *43*, 3324.

(3) Mao, H.; Luchette, P. An integrated laser-tweezers instrument for microanalysis of individual protein aggregates. *Sens. Actuators, B* **2008**, *129*, 764.

(4) Pandey, S.; Kankanamalage, D. V. W.; Zhou, X.; Hu, C.; Isaacs, L.; Jayawickramarajah, J.; Mao, H. Chaperone-Assisted Host–Guest Interactions Revealed by Single-Molecule Force Spectroscopy. *Journal of the American Chemical Society* **2019**, *141*, 18385.

(5) Pokhrel, P.; Ren, K.; Shen, H.; Mao, H. Mechanical Stability of DNA Corona Phase on Gold Nanospheres. *Langmuir* **2022**, *38*, 13569.

(6) Mills, J. B.; Vacano, E.; Hagerman, P. J. Flexibility of single-stranded DNA: use of gapped duplex helices to determine the persistence lengths of poly(dT) and poly(dA). *J. Mol. Biol.* **1999**, *285*, 245.

(7) Record, M. T. J.; Anderson, C. F.; Lohman, T. M. Thermodynamic analysis of ion effects on the binding and conformational equilibria of proteins and nucleic acids: the roles of ion association or release, screening, and ion effects on water activity. *Quart. Rev. Biophys.* **1978**, *11*, 103.

(8) Do, N. Q.; Chung, W. J.; Truong, Thi Hong A.; Heddi, B.; Phan, A. T. G-quadruplex structure of an anti-proliferative DNA sequence. *Nucleic Acids Research* **2017**, *45*, 7487.

(9) Jawad, B.; Poudel, L.; Podgornik, R.; Steinmetz, N. F.; Ching, W.-Y. Molecular mechanism and binding free energy of doxorubicin intercalation in DNA. *Physical Chemistry Chemical Physics* **2019**, *21*, 3877.

(10) Tran, P. L. T.; Mergny, J.-L.; Alberti, P. Stability of Telomeric G-quadruplexes. *Nucleic Acids Res.* **2011**, *39*, 3282.

(11) del Villar-Guerra, R.; Trent, J. O.; Chaires, J. B. G-Quadruplex Secondary Structure Obtained from Circular Dichroism Spectroscopy. *Angewandte Chemie International Edition* **2018**, *57*, 7171.

(12) Bonner, G.; Klibanov, A. M. Structural stability of DNA in nonaqueous solvents. *Biotechnology and Bioengineering* **2000**, *68*, 339.
